# Understanding the Role of Solvent on the Growth of Zinc Oxide: Insight from Experiment and Molecular Dynamics Simulations

**DOI:** 10.1021/acs.langmuir.4c00921

**Published:** 2024-09-03

**Authors:** Sherif Okeil, Sahar Rabet, Gerardo Valadez Huerta, Gabriele Raabe, Georg Garnweitner

**Affiliations:** † Institute for Particle Technology, Technische Universität Braunschweig, Volkmaroder Str. 5, 38104 Braunschweig, Germany; ‡ Center for Research Initiative for Supra-Materials, Shinshu University, 4-17-1 Wakasato, Nagano 380-8553, Japan; § Laboratory for Emerging Nanometrology, Technische Universität Braunschweig, Langer Kamp 6A, 38106 Braunschweig, Germany; ∥ Pharmaceutical Analytical Chemistry Department, Faculty of Pharmacy, Ain Shams University, Abbassia, Cairo 11566, Egypt; ⊥ Institut für Thermodynamik, Technische Universität Braunschweig, Hans-Sommer-Str. 5, 38106 Braunschweig, Germany

## Abstract

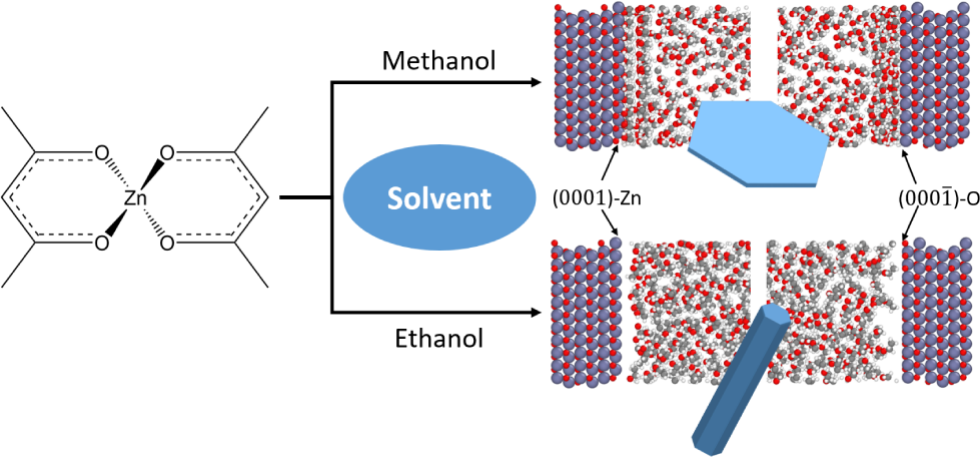

The controlled synthesis of nanoparticles with tailored shapes and morphologies has garnered significant attention, driven by the ever-growing demand for advanced materials with defined properties. In nanoparticle formation, various parameters influence the final product, and among these, the solvent plays a pivotal role, as it constitutes the major component of the reaction medium. In this work, the critical role of solvents in controlling the growth of zinc oxide (ZnO) nanoparticles was investigated, with a focus on simple primary alcoholic solvents as the reaction medium. A model reaction based on the direct solvolysis of anhydrous zinc acetylacetonate was employed to probe the influence of different primary alcohols, specifically methanol, ethanol, and their mixture. A substantial difference in the preferential growth direction of the ZnO nanocrystals in methanol and ethanol was observed through XRD and was further proven through TEM. Thereby, in ethanol, a preferential growth in the [001] direction was observed, resulting in short nanorods as primary particles, while this growth was inhibited in methanol, leading to platelet- or sheet-like primary particles. To unravel the underlying mechanisms responsible for the observed solvent-dependent variations, molecular dynamics (MD) simulations were employed using an optimized interface force field to model the ZnO-alcohol interaction. These simulations provide valuable insights into the preferential adsorption of the solvent molecules onto the polar (0001) and (0001̅) and nonpolar (101̅0) ZnO surfaces, shedding light on the fundamental interactions driving the shape control phenomenon. Essentially, the experimental observations on primary particle morphology could be explained well by the adsorption behavior determined by the MD simulations. Furthermore, this report provides an extensive comparison with various similar reaction systems for ZnO synthesis, deriving correlations with the findings from the model system. These insights contribute to a deeper understanding of the intricate interplay between solvent properties and nanoparticle growth, offering a valuable toolkit for designing and optimizing the synthesis of ZnO nanoparticles with specific shapes and functionalities.

## Introduction

Zinc oxide (ZnO) has emerged as a versatile nanomaterial with widespread applications in various fields, including electronics, optoelectronics, catalysis, and sensors, owing to its exceptional electrical, optical, and mechanical properties.^1−3^ Over the years, significant research has been conducted on the synthesis of ZnO nanostructures in diverse organic media due to the facile control over morphology and size achievable through solvothermal processes.^4,5^ However, despite numerous studies on ZnO synthesis, the role of the solvent, which constitutes the major component of the reaction system, has not been thoroughly explored. Only a limited number of works have systematically investigated the influence of the solvent on ZnO growth;^5−10^ thus, more investigation is required for a deeper understanding of the solvent effect for different reaction systems. While in the literature, some differences were observed for the primary particles of the ZnO products obtained from the synthesis in methanol and ethanol, these differences and the underlying reasons have not yet been investigated in depth. Cheng and Samulski^11^ observed different ZnO nanorod aspect ratios formed in the synthesis of ZnO from zinc acetate dihydrate and sodium hydroxide in methanol and ethanol as solvents.^11^ In that study, the authors observed different growth rates of the ZnO nanorods along the *c*-axis while using methanol and ethanol as solvents but did not investigate the underlying cause further. In fact, the detailed analysis of the solvent action on the formation of nanoparticles is difficult, as for nanostructures, the affinity and density of solvent molecules to different crystal faces can hardly be experimentally elucidated. The authors stated already in 2004 that the differences in morphology “can only be verified with detailed theoretical simulations of interface-solvent interactions using known parameters, e.g., surface energies of different crystal faces, solvent properties that are dependent on the chain length of alcohols”.^11^ Ayudhya et al.^6^ investigated the influence of different groups such as glycols, alcohols, and *n*-alkanes on the formation of ZnO from anhydrous zinc acetate, where it was found that with increasing chain length of the used alcohol, i.e., with decreasing polarity of the alcohol, the aspect ratio of the formed ZnO nanorods increased. Methanol and ethanol, however, were not subject of their studies.^6^ Motelica et al.^7^ used zinc acetate dihydrate as a precursor and investigated a long series of alcohols including methanol and ethanol at low temperature. For methanol, “rounded shape” ZnO particles were obtained, while “flower-like agglomerates” were observed from the reaction in ethanol without further statement on the particle shape in these agglomerates.^7^ Ramya et al.^8^ also investigated a series of alcohols including methanol and ethanol on the growth of ZnO from zinc acetate dihydrate and sodium hydroxide where mainly spherical particles from methanol and a mixed population of spherical and rod-shaped nanoparticles from ethanol were reported.^8^ Furthermore, Šarić et al.^9^ used zinc acetylacetonate monohydrate as a precursor and investigated its solvolysis in ethanol, 1-propanol, 1-butanol, 1-pentanol, and 1-octanol, where mainly ZnO nanorods in the form of aggregates were obtained, except for 1-butanol, in which the particles were rather isotropic. The authors attributed this unusual observation to the strong solvent-surface interactions, especially in the case of 1-butanol, resulting in the remarkable nonpreferential growth of ZnO compared to the remaining alcohol series.^9^

The rational design of ZnO nanostructures remains a challenge, as the properties of the synthesis product heavily rely on the complex interplay of various factors, with the solvent playing a crucial, yet relatively unexplored role. Therefore, our study aims to gain deeper insights into the solvent’s influence on ZnO growth is essential for fine-tuning the synthesis process and predicting the morphology of ZnO nanostructures with precision.

Previous studies that attempted to explore the role of the solvent in ZnO synthesis often employed hydrous precursor materials^7,9,12,13^ or a base as additive,^8,11^ where the presence of water or a base in the reaction medium could still exert an influence on the final outcome. To circumvent this ambiguity, our work utilized anhydrous zinc acetylacetonate as the model precursor to systematically investigate the impact of simple primary alcohols, namely, methanol and ethanol, as well as their mixture, on the growth of ZnO nanostructures in solvothermal processes. By excluding the influence of other factors, we aimed to achieve a comprehensive understanding of the solvent’s effect on the evolution of ZnO nanostructures.

ZnO has a hexagonal wurtzite-type crystal structure with faces of different polarity, which result in varied binding affinities of the solvents.^14^ The ordering of the solvents at the surface in turn has an influence on the nanoparticle growth.^15^ Several computational methods have been employed in the literature to gain a molecular level understanding of the interactions of solvents with ZnO nanoparticles. Šarić et al.^9^ accompanied their experimental work by DFT simulations and the implicit SMD polarizable continuum solvation model to study the interaction energies of different alcohols with ZnO monomers and dimers as well as small ZnO clusters, though due to the size limitation of the DFT simulations, their study did not differentiate the surface interactions of the alcohols with polar and nonpolar faces of a ZnO crystalline structure. Kiss et al.^16^ studied the adsorption of methanol on ZnO by combining experimental spectroscopy studies and DFT simulation, though only focusing on the (101̅0) surface. Dehmani et al.^17^ studied the adsorption behavior of phenol on ZnO by combining DFT and molecular dynamics (MD) simulations, though the MD study only covered the configuration of phenol on the ZnO(101) plane. Furthermore, classical force fields for MD simulations are usually designed to describe bulk phases and thus not parametrized to correctly describe interfacial interaction, e.g., of solvent-surface interactions. Therefore, in recent work, force fields were parametrized to DFT interaction energies of small molecules with ZnO surfaces to yield specific molecular models for MD studies on hydrated ZnO surfaces^18^ and the interaction of biomolecules with the ZnO(101̅0) facet.^19^ In a previous study,^20^ we presented the parametrization of an interfacial force field for MD simulation based on ab initio calculations for the methanol|ZnO interface. We extend here our work to the ethanol|ZnO surface. These specific interface force fields are then used to perform MD simulations to gain a comprehensive understanding of the solvent’s interaction with the nonpolar (101̅0) as well as the polar (0001) and (0001̅) ZnO surfaces and their effect on ZnO growth. This integrated approach allowed us to cross-validate the results from experiment and simulation and to corroborate the experimental data with atomistic-level insights, thereby reinforcing our conclusions. By shedding light on the intricate interplay between the solvent and ZnO growth, this study lays the groundwork for more precise control and prediction of ZnO nanostructure synthesis in organic media.

The insights derived from this study are expected to serve as a foundational stepping stone in predicting ZnO growth in more complex alcoholic media. Additionally, to gain further clarity on the influence of organics present in the precursor, additional experiments were conducted using different ZnO precursors. These experiments revealed intriguing findings concerning the effect of the precursor type, enhancing our comprehension of the overall ZnO growth process.

## Methods

### Solvothermal Synthesis of ZnO Nanostructures

For the solvothermal synthesis of ZnO, a specified amount of a ZnO precursor (anhydrous zinc acetylacetonate (Zn(acac)_2_) (Merck), zinc acetylacetonate hydrate (Zn(acac)_2_·*x*H_2_O) (Sigma-Aldrich), anhydrous zinc acetate (Zn(OAc)_2_) (Sigma-Aldrich), or zinc acetate dihydrate (Zn(OAc)_2_·2H_2_O) (Sigma-Aldrich)) was dissolved in 25 mL of methanol (HPLC grade, ≥99.8%, Fischer Scientific), ethanol (HPLC grade, ≥99.8%, Sigma-Aldrich), or a 1:1 v/v mixture of both alcohols. The used solvents had negligible amount of water according to the suppliers’ specifications and thus were used as received. To ensure complete dissolution, or formation of a homogeneous dispersion of the precursor in the solvent in case of partial dissolution, the precursor was stirred for 1 h with the solvent before being transferred to the autoclave (Parr Instruments). The autoclave was then placed in an oven at the desired temperature (100 or 200 °C) for 4 h. In a separate synthesis, ZnO was prepared using alkaline solvolysis. For alkaline solvolysis reactions, a 1:1 molar ratio of Zn precursor to sodium hydroxide (NaOH) was weighed and mixed with 25 mL of pure alcohol (methanol or ethanol) at room temperature and finally transferred to the autoclave to be heated at 200 °C for 4 h. The obtained product was centrifuged at 8000 rpm for 5 min and washed three times with ethanol, then it was left to dry in the ambient atmosphere to finally obtain a white powder.

### Characterization of the Obtained Products

The obtained product was characterized with X-ray diffraction (XRD) using an Empyrean diffractometer (Malvern Panalytical Ltd.) at a wavelength of 0.154 nm with copper K_α_ radiation (Empyrean Cu LFF HR) in a range of 2Θ from 20 to 90° and a step size of 0.01° (PIXcel-3D detector, Malvern Panalytical Ltd., Malvern, United Kingdom). The obtained diffractograms were evaluated using the Scherrer equation in order to determine the crystallite size of the primary particles. For that, the reflections at 31.7° and 34.4° were used in order to determine the crystallite size in the *m*-direction ([101̅0] direction) and the *c*-direction ([0001] direction), respectively.

Scanning electron microscopy (SEM) and SEM in transmission mode (STEM) were performed on a focused ion beam (FIB) scanning electron microscope (Thermo Scientific Helios 5 UX DualBeam) with the STEM detector using 30 kV acceleration voltage. Transmission electron microscopy (TEM) was performed on a Tecnai G2 F20 TMP (FEI) at a 200 kV accelerating voltage.

## Simulations

### Molecular Modeling

In our previous computational study,^20^ a new genetic algorithm was presented that enables the parametrization of interfacial force fields for MD simulation based on DFT simulations to allow for an accurate description of the nonbonded interaction energies and forces at fluid|solid interfaces. There, the algorithm was applied to develop an interfacial force field for the methanol|ZnO interface. MD simulations using the developed interfacial force field model reproduced the most stable adsorption configurations of single methanol molecules on both the nonpolar (101̅0) surface and the polar (0001) Zn surface of ZnO. These calculations were in good agreement with experimental data and ab initio simulation results from literature. Following the same procedure as described in this earlier work, an ethanol|ZnO interfacial model was developed in this work.

The interfacial models that describe the nonbonded interactions between the different atom types of the alcohol molecules and the ZnO atoms are based on the Alrich–Penco–Scoles (APS) force field with the following functional form:
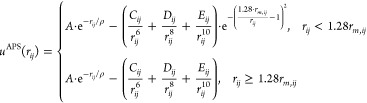


This requires the optimization of 5 parameters (A, ρ, C, D, E) for each atom pairing, i.e., for the different atom types of methanol (C, H_C_, O, H_O_) and ethanol (C_1_, C_2_, O, H_O_, H_C1_, H_C2_) with the Zn and O_ZnO_ atom types of the ZnO slab. Using the genetic algorithm, these parameters were optimized to reproduce interaction energies and forces from ab initio simulations using the DFT method with the Perdew–Burke–Ernzerhof (PBE) functional^21^ with D3 dispersion correction.^22^ Details on the DFT simulations and on the parametrization procedure using the genetic algorithm are provided in ref (20).

The optimized parameters A, ρ, C, D, and E as well as the resulting distances r_m_ of minimum energy for all atomic APS pair potentials of the interfacial methanol|ZnO and ethanol|ZnO force fields are given in the Supporting Information. There, in Figures S1 and S2, we also provide a comparison of calculated forces and energies using the newly optimized ethanol|ZnO model with ab initio data. Table 1 provides a comparison between MD results using our optimized interface force fields with ab initio (DFT) data from the literature for the adsorption energy of single methanol and ethanol molecules on the ZnO surface.

**Table 1:** Adsorption Energies of a Single Alcohol Molecule Attached to the (101̅0) ZnO Surface at 298.15 K: Comparison of MD Results using the Interfacial Force Field in Comparison with Literature Data (DFT)^a^

	property	interfacial-FF	literature
methanol	in eV	–0.938(46)	–1.0,^23^ −1.14^24^
	in eV	–0.99	
ethanol	in eV	–0.9972(66)	
	in eV	–1.03	–0.92,^25^ −1.18^26^

a The value *e*_ads,min_ resulted from an energy minimization procedure.

The comparison with DFT data well reflects that our interfacial force fields are able to reproduce the ab initio results for single molecule adsorption, whereas it allows us to extend the study of the adsorption behavior to the larger scale. With this, we are able to study bulk effects in the liquid phase such as hydrogen bonding, orientation toward the surface in the bulk, competing behavior in mixtures, etc.—which are topic of our present study and not feasible with DFT simulations.

Whereas the interactions between the molecules of the fluid phase and the ZnO surface are described by the optimized interfacial force fields, the bulk alcohol phases are modeled by the OPLS-AA force field. Thereby, all OPLS-AA parameters for methanol and ethanol were generated using the LigParGen web server.^27−29^ The interactions between Zn and O_ZnO_ within the ZnO slab were modeled by the ab initio based force field by Wang et al.^30^ It should be noted that all Coulombic interactions are calculated based on the constant partial charges given by the original bulk phase force fields.

### MD Simulations

Using the interfacial methanol|ZnO and ethanol|ZnO force fields, MD simulations were carried out to examine the interaction of ethanol and methanol molecules with polar and nonpolar ZnO surfaces. All simulations were conducted using the LAMMPS^31,32^ simulator. OVITO^33^ was used for the visualization while in-house python tools were used for the postprocessing of the simulations.

**Figure 1:**
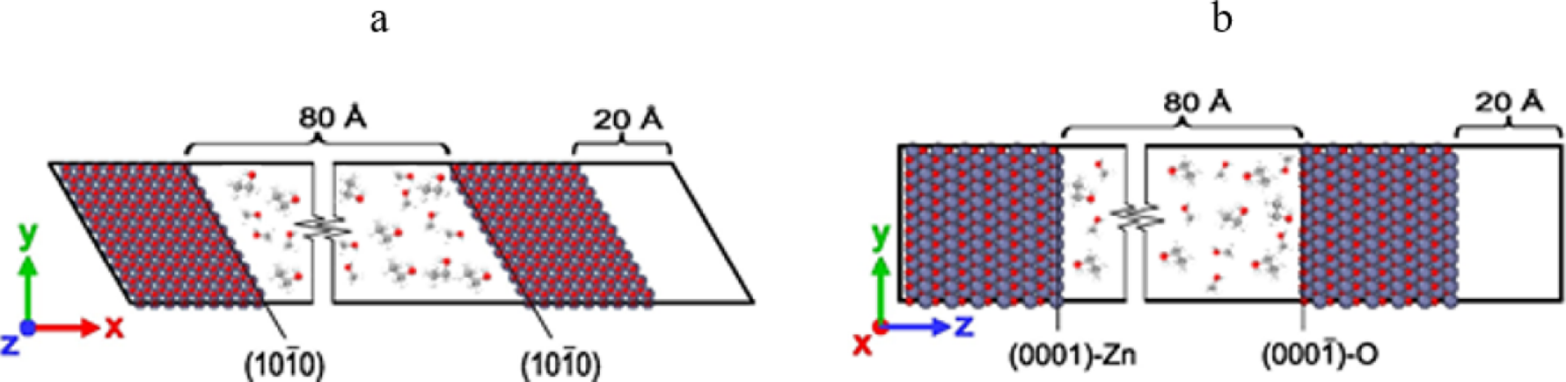
Illustration of the simulated systems with slabs having (a) nonpolar and (b) polar surfaces. The 80 Å gap was filled with the liquid phase.

The simulations included two ZnO slabs and a liquid phase between them, i.e., methanol, ethanol, or 50 mol % mixture of ethanol/methanol. Two wurtzite hexagonal structures of ZnO were considered with lattice constants of *a* = 3.26 Å and *c* = 5.26 Å.^20^ The first structure included two same slab surfaces with the (101̅0) nonpolar surfaces (shown in Figure 1a) and the second structure included two polar slabs, on one side a (0001)-Zn face in contact with the liquid phase, and on the other side the (0001̅)-O face (depicted in Figure 1b). The simulation cell was periodic in the *x*, *y*, and *z* directions. The gap between two slabs filled with the liquid phase has a length of 80 Å; additionally, a vacuum gap of 20 Å gap between the ZnO slabs was inserted to prevent the interaction between the system and its periodic image.

The number of ethanol and methanol molecules in each simulation was defined to yield the saturated liquid densities of the alcohols, determined by their reference equation of state.^34^ The gap between two ZnO slabs was filled with ethanol and/or methanol molecules, considering their density at the simulation conditions. Due to the strong adsorption of methanol to the ZnO surfaces, some “gas bubbles”, or rather, voids were formed in the bulk phases of the methanol-containing simulation systems. Therefore, for these systems, we increased the number of molecules in the bulk up to 30% of the original value to get a homogeneous bulk density; this did not affect the results for adsorption behavior close to the surfaces. The number of molecules in each simulation is summarized in Table S3. A 12.5 Å cutoff was employed for the Lennard-Jones and Coulomb interactions of the liquid phase (methanol and/or ethanol), while an 8 Å cutoff was considered for the interaction of liquid and the surface. The Ewald solver with an accuracy of 1.0 × 10^–5^ relative error on the forces was used for long-range electrostatic interactions. A 0.5 fs time step was considered for all simulations. Moreover, all simulations were performed for two kinds of slab structures (with polar and nonpolar surfaces, respectively) and two temperatures (100 and 200 °C). The SHAKE^35^ algorithm with an accuracy tolerance of 1.0 × 10^–4^ was used for constraining the bonds between hydrogen atoms and other atoms in each molecule.

Each simulation in the bulk phase was started with an energy minimization (with conjugate gradient (cg) algorithm) of the bulk phase followed by an equilibration phase with the NVT ensemble, considering a 3.5 Å gap between the bulk and slab surfaces. All NVT simulations in this work were carried out using a Nosé–Hoover^36,37^ thermostat with three thermostats in the particle thermostat with the temperature damping parameter of 100 time steps. The equilibrated bulk phase was then located between the two slabs. In this phase of simulations, the ZnO slabs were frozen with zero force on them. Each simulation started with a minimization, followed by an equilibration in the NVT ensemble for 1 ns. After equilibration of the system, the production run was conducted for 500 ps, and the trajectory was saved every 100 timesteps for postprocessing.

## Results and Discussion

### Products Obtained from the Solvothermal Synthesis of ZnO in Simple Primary Alcohols

For the synthesis of ZnO in the simple alcohols, methanol and ethanol, Zn(acac)_2_ was chosen as the precursor in order to keep the system simple and to eliminate any other possible factors that could influence the ZnO growth. Moreover, anhydrous Zn(acac)_2_ was selected as a precursor for the initial studies to ensure the exclusion of water from the reaction system. The precursor was mixed with the alcohol to be investigated as the solvent and heated in the autoclave at the set reaction temperature, which ensures that no other substances may exert an influence on the growth of the ZnO. The synthesis was performed in the pure solvents such as methanol and ethanol as well as a 1:1 v/v mixture of methanol and ethanol.

**Figure 2:**
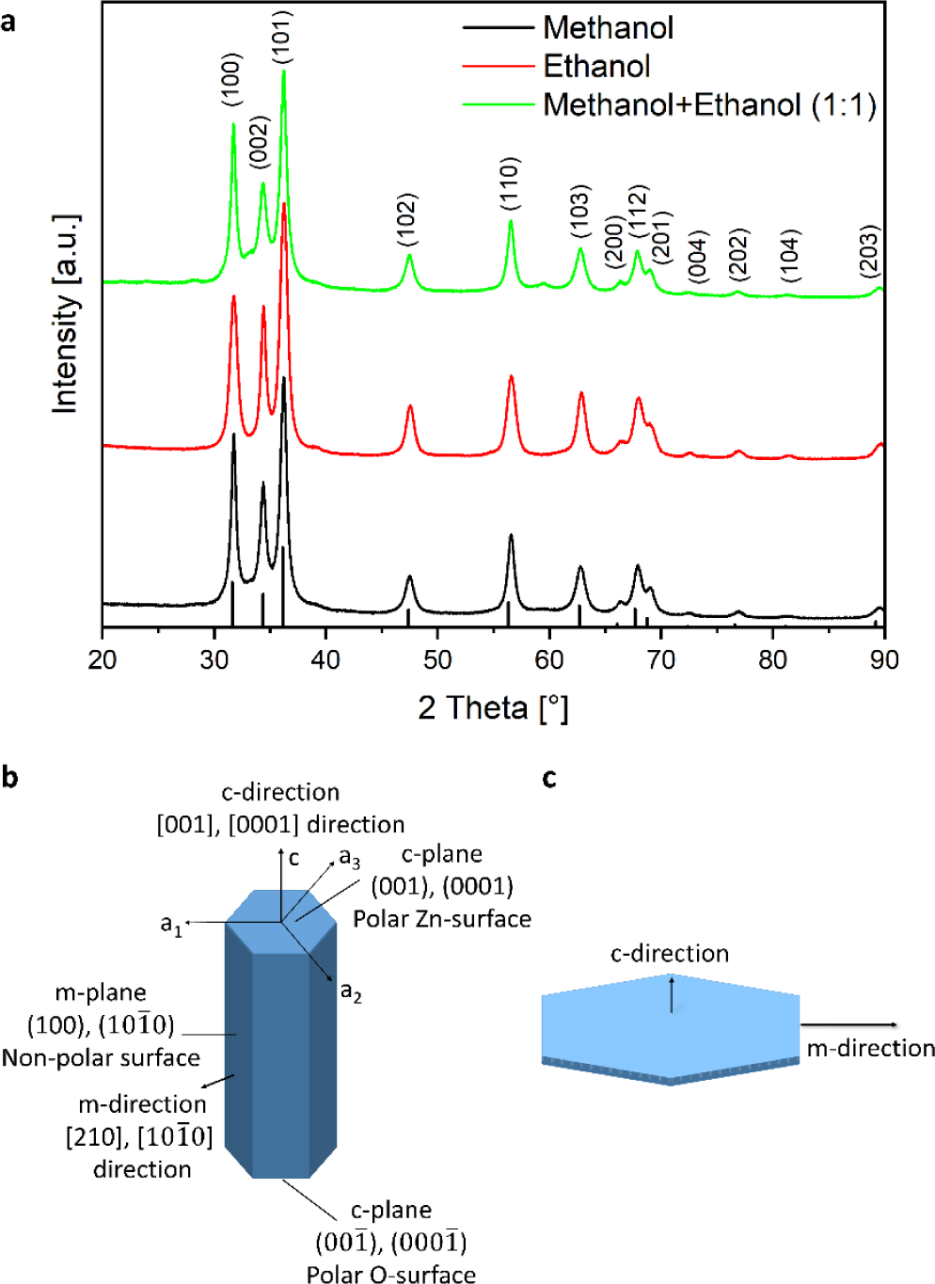
(a) X-ray diffractograms of the products obtained from the solvothermal synthesis using anhydrous Zn(acac)_2_ as a precursor in pure methanol, pure ethanol, and a 1:1 (v/v) mixture of methanol and ethanol at 200 °C showing the formation of wurtzite-phase ZnO. The reflections of the reference are presented as black lines at the bottom of the diagram (ICSD No. 98-006-5122). Schematic illustration showing the hexagonal crystal structure of wurtzite-phase ZnO with the main crystal planes and possible growth directions where (b) a preferential growth in the *c*-direction ([001] or [0001] direction) results in a rod-shaped morphology while (c) a preferential growth in the *m*-direction ([210] or [101̅0] direction) results in a platelet- or sheet-like structure. The longer arrows represent the preferential growth direction of the crystal.

The reaction mechanism of zinc acetylacetonate was previously elucidated in the literature with the alcohols, 1-butanol and isobutanol.^38^ The analogous reaction mechanism was expected with methanol and ethanol, which was proven through performing ^13^C NMR measurements of the supernatant obtained from the product dispersion after removing the formed ZnO nanoparticles through centrifugation (Figure S3a,b). The reaction does not proceed via a standard hydrolytic sol–gel mechanism, but via a nonaqueous mechanism involving alcoholytic C–C cleavage of the acetylacetonate resulting in the formation of methyl acetate or ethyl acetate esters along with acetone as side products. Water is not formed in these reactions so that this reaction proceeds in a completely nonaqueous manner. The products obtained from the different syntheses were characterized via XRD, and wurtzite-phase ZnO (zincite; ICSD database, no. 98-006-5122) was found. While all products showed wurtzite phase ZnO, a substantial difference in the intensity ratios of the (100) and (002) reflections could be observed (Figure 2a) which relates to differences in the crystallites obtained in the different alcohols. In comparison to methanol, the product of the ethanol-based synthesis shows a significant increase in the (002) reflection, whereas the product obtained from the 1:1 methanol: ethanol mixture shows a similar XRD pattern.

**Figure 3:**
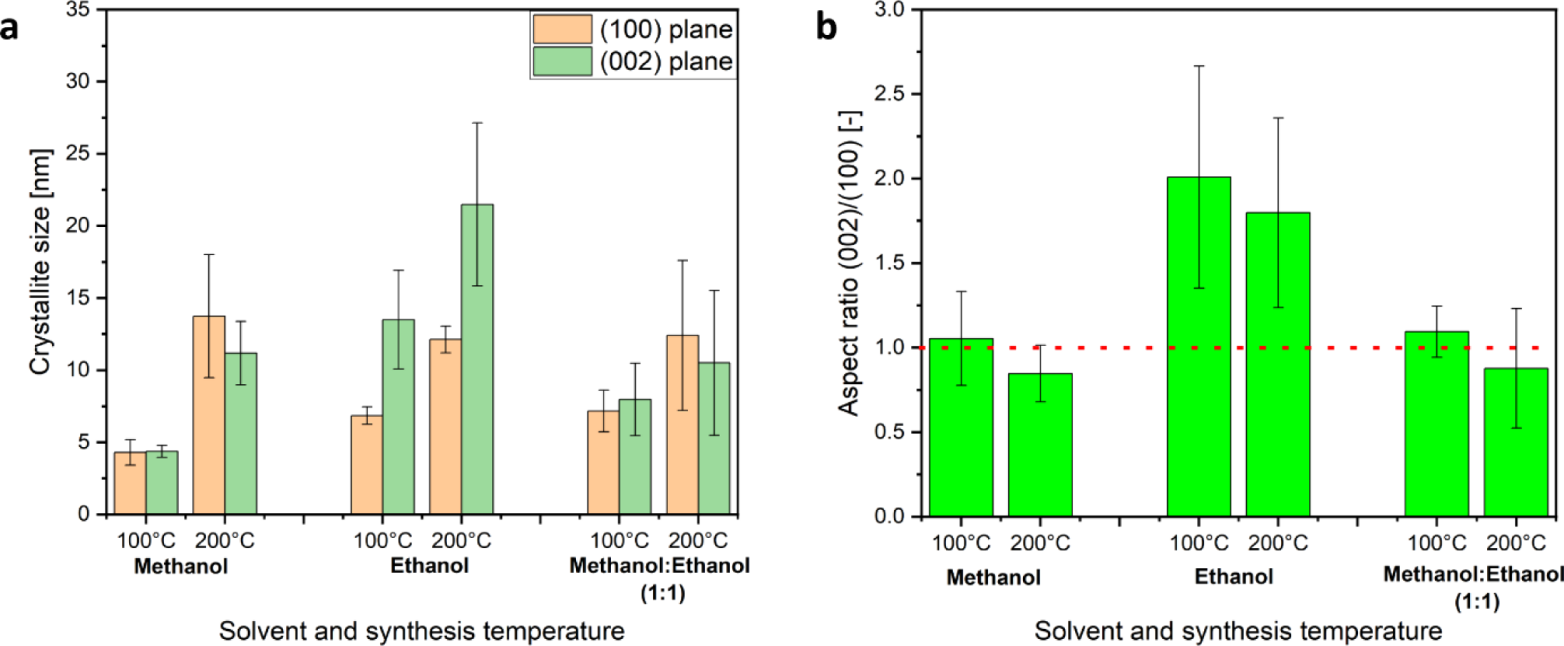
Bar chart showing (a) the crystallite size and (b) the corresponding aspect ratio calculated via the Scherrer equation from the (100) and (002) reflections of the XRD data measured for the products from syntheses at 100 and 200 °C using anhydrous Zn(acac)_2_ as the precursor in the different solvents methanol, ethanol, and the 1:1 methanol: ethanol solvent mixture.

The (100) plane (representative for the *m*-plane or {101̅0} planes in the hexagonal system) represents the nonpolar facets, while the (002) plane (representative to the *c*-plane or {0001} planes in the hexagonal system) represents the polar facets of the zincite crystal (Figure 2b). Thus, these reflections were used to calculate the dimensions of the formed ZnO primary particles in the *m*- and *c*-directions using the Scherrer equation, thus giving an indication of the shape of the particles. While an elongation of the crystal in the *c*-direction corresponds to nanorod-shaped particles, an elongation in the *m*-direction relates to platelet-like or nanosheet structures (Figure 2c). By evaluation of the XRD data collected for the products obtained from different syntheses at 100 and 200 °C, it was found that using ethanol as a solvent resulted in particles that are elongated in the *c*- or [0001] direction. For methanol an inverse behavior was observed, as the primary particles tended to be elongated along the *m*- or [101̅0] direction, which would suggest a tendency toward a two-dimensional platelet-like structure (Figure 3). Thus, for methanol, a “reversal” of the preferential growth direction of the ZnO nanocrystals is observed in comparison to ethanol, leading to a much lower (002)/(100) aspect ratio with a value below 1 for the synthesis at 200 °C (Figure 3b). A mixture of both solvents showed results similar to those using methanol alone as a solvent (Figure 3), which suggests the dominant role of methanol in the solvent mixture in determining the ZnO growth.

**Figure 4:**
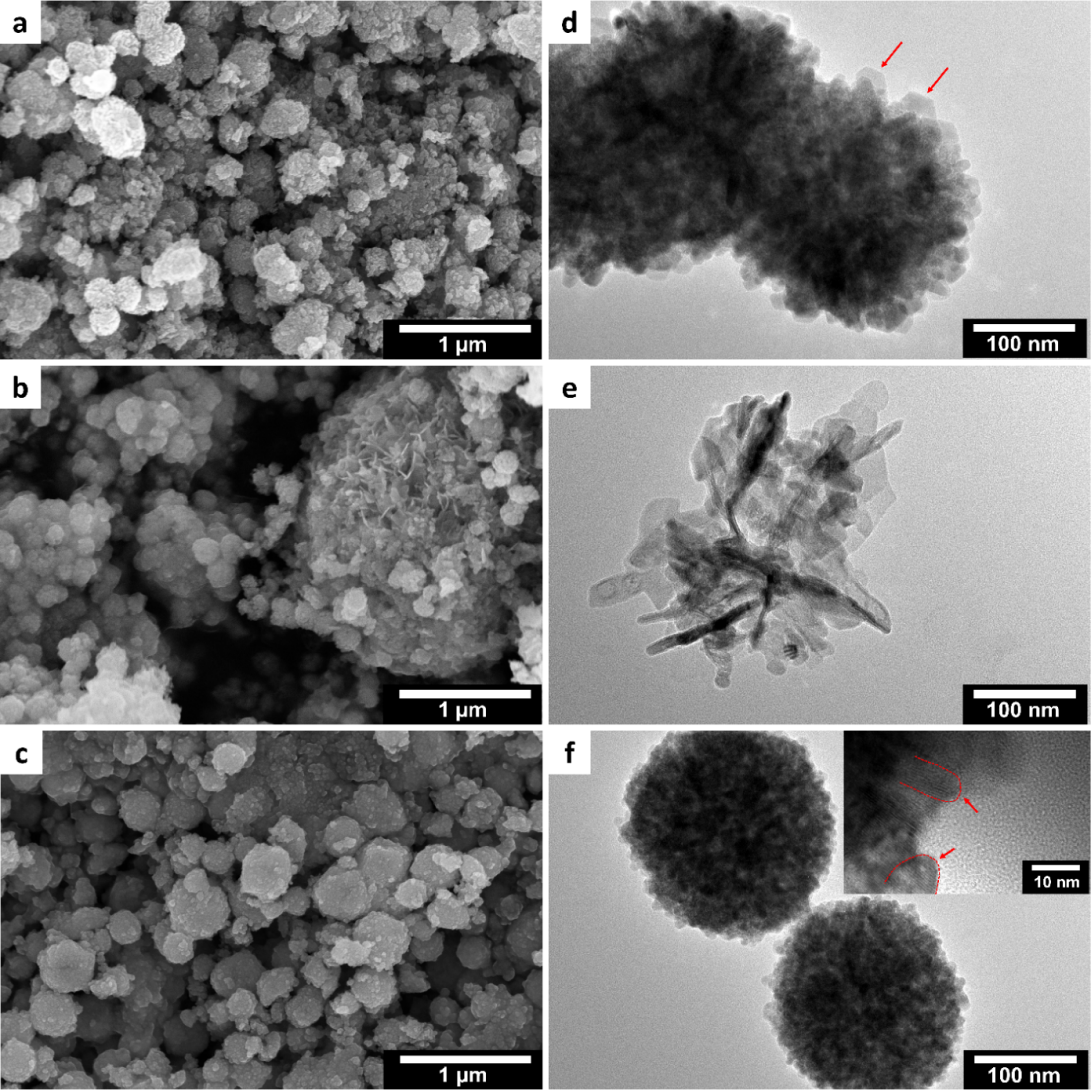
SEM and TEM images of the ZnO formed from anhydrous Zn(acac)_2_ at 200 °C in (a, d) pure methanol (the red arrows point toward positions where the platelet structure at the border of the spherical particle aggregates is visible), (b, e) 1:1 methanol:ethanol solvent mixture, and (c, f) pure ethanol. The inset shows the edge of the spherical particle aggregate at higher magnification. The red lines indicate the visible borders of the primary particles at the edge of a spherical particle aggregate.

Electron microscopy was further performed to investigate the obtained ZnO structures. Analysis using SEM showed the formation of spherical aggregates in methanol and ethanol as reaction media, while for the solvent mixture irregular aggregates resulted, which complicated the investigation of the formed primary particles for all systems (Figure 4a–c). Investigations using TEM in which we searched for some broken aggregates confirmed the mostly two-dimensional platelet morphology in the case of methanol as the solvent, which can be clearly seen at the border of the aggregated structures (Figure 4d). Similarly, the product obtained from the solvolysis of Zn(acac)_2_ in the 1:1 methanol: ethanol solvent mixture clearly shows the higher tendency toward two-dimensional platelet or sheet-like structures although some rod-shaped structures can also be observed (Figure 4e). When using ethanol as the solvent, some elongated particles could be identified at the border of the spherical aggregates (Figure 4f), which corroborates the previous XRD results.

In literature, ZnO is known to form rod-shaped particles due to the preferential growth of ZnO along the [001] direction.^9,39,40^ As in the literature, mainly alkaline hydrolysis is used for the solvothermal synthesis of ZnO, and the role of the solvent cannot be elucidated, as other factors could superimpose the influence of the solvent on the ZnO crystal growth. Furthermore, the use of hydrated precursors introduces water into the reaction system, which can also have an influence on the ZnO growth. For example, Ramya et al.^8^ investigated the influence of a series of alcohols on the ZnO growth where the difference between using methanol and ethanol as the solvent was mainly in the larger aspect ratio with the increasing chain length of the used alcohol. Thus, mainly more rod-shaped ZnO particles were observed upon using ethanol as a reaction medium while “less rod shaped” or spherical particles were observed with methanol as the reaction medium,^8^ which shows the tendency of methanol to inhibit the ZnO growth along the *c*-axis. However, this is the first time to observe the reversal of the growth direction of ZnO; as other factors (such as traces of water and other reactants such as NaOH) were excluded in this study, the role and influence of methanol on the ZnO growth are clearly demonstrated.

The formation of secondary structures (aggregates) as observed in the SEM and TEM images is most probably due to the type of precursor used, where the organics are responsible for the formation of bridges between the primary particles, resulting in these secondary structures. Typically, the nonaqueous synthesis in organic media results in the formation of agglomerates unless stabilizing surfactant species are present in the reaction mixture.^41^ According to Khokhra et al.,^42^ the aggregation of ZnO nanomaterials can be induced by hydrogen bonding between the surface hydroxyl groups of the primary particles and the solvent molecules which results in bridge formation between the primary particles leading to their aggregation. Analysis of the formed products by IR spectroscopy and thermogravimetric analysis (TGA) (Figures S4 and S5) reveals the presence of organics in the final product even after washing, with a mass loss of about 6% in the TGA for the products obtained from Zn(acac)_2_ suggesting the presence of organics within the formed secondary structures. As the influence of the used solvent and formed organic byproducts on aggregate formation is highly complex, the following discussion concentrates on the influence of the different solvents on the morphology of the primary particles as determined from the XRD and the microscopic investigation. In order to understand the ZnO primary particle growth in these alcohols and the alcohol mixture, the specific interactions between the alcohols and the different ZnO surfaces were analyzed by MD simulations.

### MD Results

To gain insight into the adsorption behavior of the alcohols on the polar and nonpolar ZnO surfaces, the concentration profiles of the center of mass of methanol and ethanol molecules, and the number density profiles of methanol and ethanol as a function of their distance from the ZnO surface were analyzed, in both the pure fluids and the mixture, at 100° and 200 °C. For the systems with nonpolar surfaces, symmetrical concentration profiles are expected as the surfaces on the left and right of the simulation box are identical (101̅0). Though, in the system with polar surfaces, the surface on the left is (0001)-Zn, and the surface on the right is (0001̅)-O that show different adsorption behavior for the alcohols as will be discussed in detail in the following sections.

### Adsorption of Methanol on the Polar and Nonpolar Surfaces

**Figure 5:**
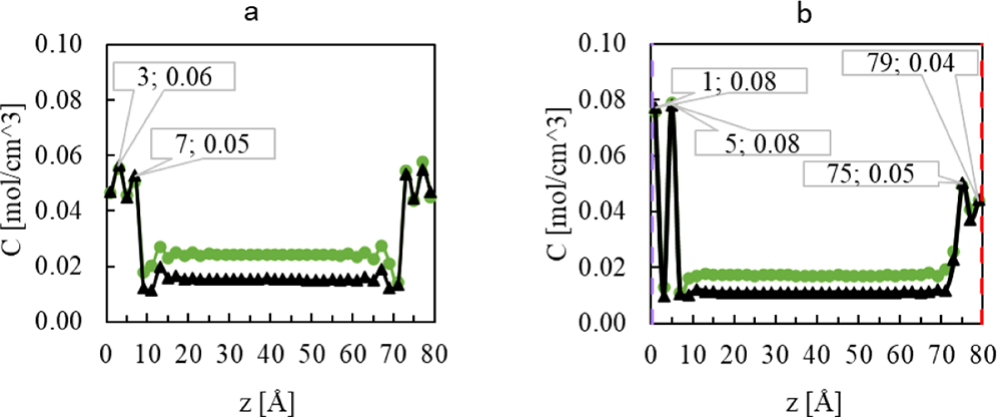
Concentration of methanol molecules at (a) nonpolar (101̅0)-ZnO and (b) polar ZnO surfaces at 100 °C (green circle marker) and 200 °C (black triangle marker). In the polar system, the left side is (0001)-Zn surface (purple dashed line), and the right side is the (0001̅)-O surface (red dashed line).

The concentration profiles of pure methanol at both temperatures on the nonpolar ZnO surfaces are shown in Figure 5a, and on the polar (0001)-Zn and (0001̅)-O surfaces in Figure 5b. On all surfaces, we observe a significant binding affinity of methanol with two distinct peaks in the concentration profile, indicating the formation of two adsorption layers. However, the shape and characteristic of the concentration curves differ for the polar and nonpolar surfaces.

The adsorption concentration of methanol at the nonpolar surface in Figure 5a shows two maxima at 3 and 7 Å distance from the surface. As illustrated in Figure 5b, the methanol molecules have a higher accumulation trend toward the Zn surface with the two maxima at 1 and 5 Å distance from both surfaces. Based on these findings, it is reasonable to assume that the methanol molecule completely covers the Zn-terminated ZnO surface, preventing the ZnO growth in this direction. On the other hand, the adsorption concentration of methanol is similar at both the O-terminated ZnO surface and at the (101̅0) nonpolar surface, indicating probable growth toward these surfaces. This observation is in agreement with the observed (200)/(001) aspect ratio of around 1 as shown also in the experiments above.

The MD simulations did not detect any temperature effect on the adsorption concentration on both nonpolar and polar surfaces, while the experiment showed a decreased (200)/(001) aspect ratio with an increased crystallite size at higher temperatures.

To gain deeper insight into the adsorption behavior of methanol on the different surfaces, the density profiles of different atoms on the surface were analyzed. The number density profiles for methanol at the ZnO (101̅0), (0001)-Zn, (0001̅)-O at 100 and 200 °C are shown in Figures S10 and S11.

**Figure 6:**
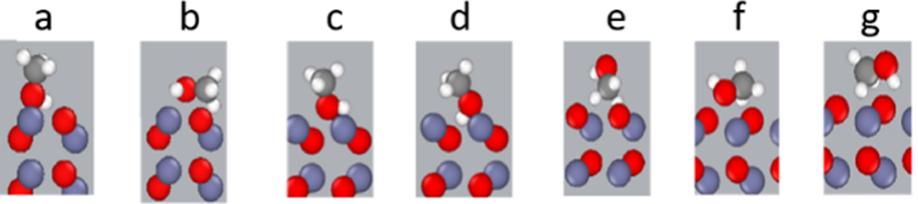
(a–g) Preferred orientations of the methanol molecules toward the different ZnO surfaces.

At nonpolar surfaces, we observe adsorptions between the oppositely charged atoms Zn on the surface and O in methanol molecules, while the H atom in the hydroxyl group tends to form a hydrogen bond with the surface oxygen (Figure 6a). This adsorption configuration was previously observed in the DFT studies to be the most stable,^16,24^ though also another adsorption configuration (Figure 6b) can be observed which might occur when the adsorption sites for the most stable configuration are already occupied.

The analysis of the density profile of methanol at the (0001)-Zn polar surface indicates a high inclination of the hydroxyl group to the surface, as shown in Figure 6c. However, a small number of molecules can be observed having an adsorption orientation similar to that in Figure 6d, in which H in the hydroxyl group forms a hydrogen bond with the surface oxygen.

At the polar (0001̅)-O surface, methanol shows the higher tendency to adsorb to this surface with the methyl groups. We can assume the adsorption configuration to be similar to that in Figure 6e–g, though with a higher probability of the adsorption configurations shown in Figure 6f,g.

**Figure 7:**
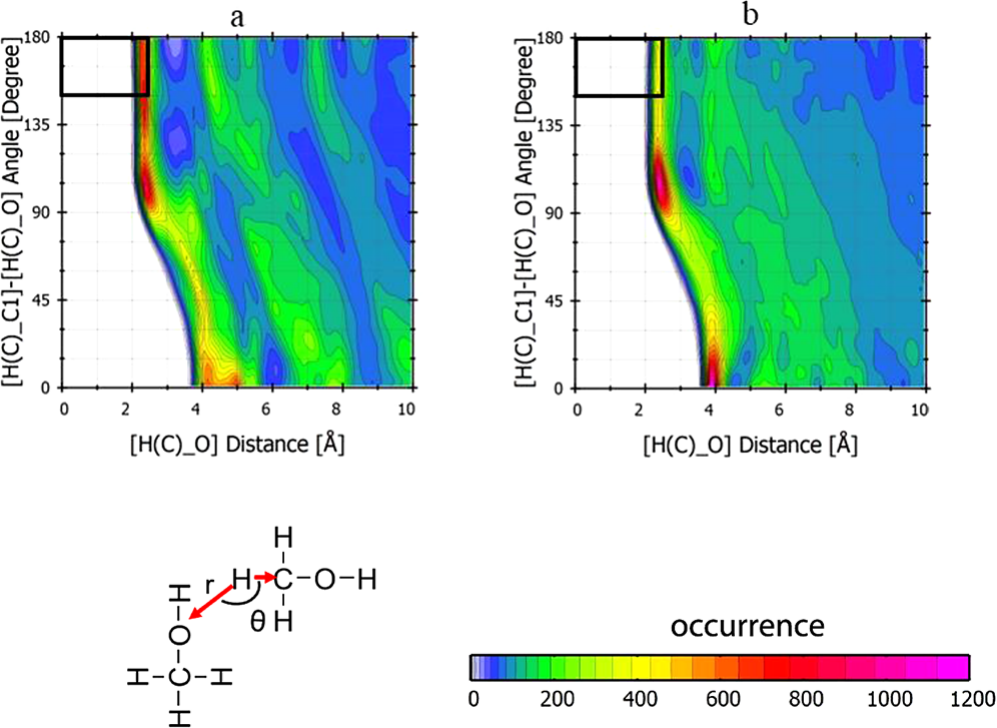
Combined distribution function of the hydrogen bond geometry between methanol molecules at the first two adsorbed layers at (a) (0001)-Zn and (b) the (0001̅)-O surfaces at 100 °C. The black squares indicate the geometrical hydrogen bond criteria.

To investigate the hydrogen bonding between the first two adsorbed layers of methanol, the combined distribution functions (CDF) of methanol molecules were calculated using TRAVIS^43,44^ for both polar surfaces. For this purpose, a region up to 9 Å distance from the surface was considered on both polar surfaces, and the CDF for the distance and angle distribution of bonding between hydrogen in the methyl group (as a hydrogen bonding donor) and oxygen in the hydroxyl group (as a hydrogen bonding acceptor) were computed (Figure 7). Figure 7a depicts the hydrogen bonding of CH(C)–O between the first two adsorbed layers at (0001)-Zn, while Figure 7b presents the same hydrogen bonding at the (0001̅)-O surface. The hydrogen bonding is identified by the combined distance/angle geometrical direction as *r*(H(C), O) ≤ 2.5 Å and 150° ≤ θ(C, H(C), O) ≤ 180°.^45−47^ As can be seen, the occurrence of hydrogen bonding at the Zn-terminated ZnO surface is higher than at the O-terminated surface, which could result from the strong orientation of the methanol molecules at the first layer as described above.

### Adsorption of Ethanol on the Polar and Nonpolar Surfaces

**Figure 8:**
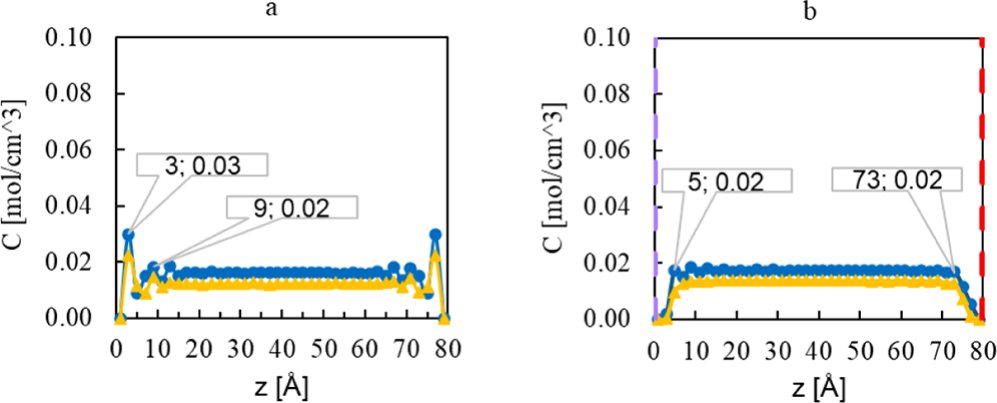
Concentration of ethanol at (a) the nonpolar (101̅0)-ZnO and (b) the polar ZnO surfaces at 100 °C (blue circle marker) and 200 °C (yellow triangle marker). In the system with polar surfaces, the left side corresponds to the (0001)-Zn surface (purple dashed line) and right side to the (0001̅)-O surface (red dashed line).

The calculated adsorption concentration of ethanol on the nonpolar ZnO surface is shown in Figure 8a. The observed layered structure for methanol adsorption also appears for ethanol with the first two maxima at 3 and 9 Å distance from the surface. The distance between the first and second peaks amounted to 4 Å for the methanol system, while it is 6 Å for the ethanol system, which could corroborate the findings of Zobel^48^ that the alcohol molecules are adsorbed to the (101̅0)-ZnO surface with hydrogen bonding in normal orientation to the surface; thus, the maxima and minima of the oscillations move further away from the surface as the alkyl chain length increases.

The adsorption concentration of ethanol on the polar surfaces is shown in Figure 8b. Here, the maximum concentration is reached at a higher distance of about 5 Å at both surfaces, which shows a low tendency of ethanol molecules to adsorb to both the (0001)-Zn surface and the (0001̅)-O surface. The higher adsorption tendency of the molecules toward nonpolar surfaces in comparison to polar surfaces is in agreement with the preferential growth of ZnO in the polar direction in the ethanol system, resulting in the high (002)/(100) aspect ratio observed in the experiment (see Figure 3). The less pronounced interactions between ethanol and ZnO compared to the methanol systems, indicated by the reduced peak heights in the concentration profiles, might also explain the stronger growth of ZnO crystals in ethanol with larger crystallite sizes in these systems. When increasing the temperature in the ethanol-ZnO synthesis, the maximum amplitude of the ethanol concentration in the adsorbed layer at the nonpolar surface was slightly decreased, which would enable an increased growth rate in this *m*-direction. This explains the lower (002)/(100) aspect ratios obtained for the ZnO particles at higher synthesis temperature, as shown in Figure 3b.

**Figure 9:**
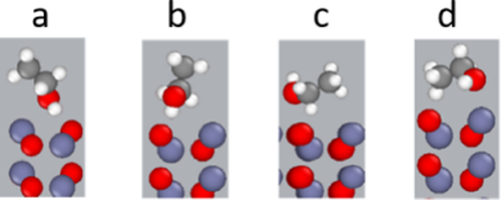
(a–d) Preferred orientations of the ethanol molecules toward the nonpolar (101̅0)-ZnO.

The number density profiles for ethanol at 100 and 200 °C at the ZnO (101̅0), ZnO (0001), ZnO (0001̅) surfaces are provided in Figures S12 and S13. They imply the presence of diverse adsorption configurations at the nonpolar (101̅0)-ZnO by approaching different hydrogens as shown in Figure 9a–d. However, the higher density of hydrogen in the hydroxyl group suggests a higher likelihood of adsorption with this functional group, as illustrated in Figure 9a,b. These configurations are similar to the most stable adsorption of ethanol determined by DFT calculation.^26^ However, as already mentioned for methanol, the adsorption behavior in our study in the fluid phase is also influenced by temperature, interaction between ethanol molecules, and site competitiveness.

In general, the interaction of the ethanol molecule with the two polar surfaces is too weak to influence the orientation of the molecules on the surface, as shown in the concentration curve (Figure 8b).

### Adsorption of the 1:1 Methanol–Ethanol Mixture on the Polar and Nonpolar Surfaces

**Figure 10:**
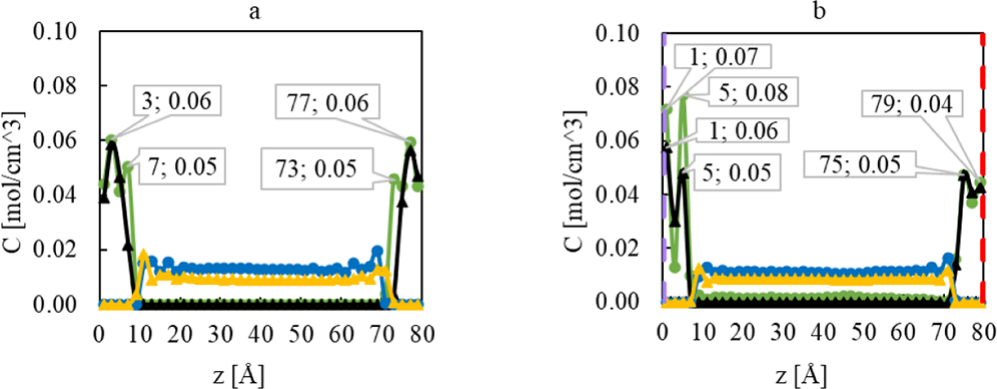
Calculated concentration of methanol and ethanol in a 1:1 molar mixture at (a) the nonpolar (101̅0)-ZnO and (b) the polar ZnO surfaces at 100 °C (methanol: green circle marker, ethanol: blue circle marker) and 200 °C (methanol: black triangle marker, ethanol: yellow triangle marker). In the system with polar surfaces, the left side is (0001)-Zn surface (purple dashed line), and the right side is the (0001̅)-O surface (red dashed line).

The concentration of the methanol and ethanol molecules at the nonpolar and polar surfaces in the 50 mol % mixture of ethanol/methanol is shown in Figure 10a,b, respectively. As can be seen, the behavior of ethanol and methanol molecules is nearly identical with the unmixed systems. The peaks of the methanol concentration at 100 °C appeared at the same distance as in the unmixed systems for both the nonpolar and polar surfaces. Thus, the accumulation of methanol at the surfaces shows the same trend as that for the pure methanol system. Therefore, the preferential growth in the mixture systems is similar to that discussed previously for the methanol system, resulting in a nearly identical (002)/(100) aspect ratio for both systems. The similarity of the behavior of the pure methanol and the mixture system can be explained by the observation of a clear separation between ethanol and methanol molecules in the mixture, where the methanol molecules accumulate completely on the surface, while the ethanol molecules avoid the surface. The distances of the maximum peaks from the surfaces for the ethanol molecules are even further increased, as the first peak appears at 11 Å.

In contrast to the pure methanol systems, however, a temperature effect on the concentration profile can be observed in the mixture. At the higher temperature of 200 °C, only one methanol peak can be seen close to the nonpolar surface, and the intensity of the methanol peaks close to the (0001)-Zn surface decreased compared to the peak height at 100 °C. This could be due to the lower number of methanol molecules in relation to the available adsorption sites on the ZnO surfaces and might explain the increased crystal growth at higher temperatures observed in the experiments as shown in Figure 3a. It should be noted that a 1:1 molar mixture was studied in the simulations, while a 1:1 (v/v) mixture was investigated in the experiment, which corresponds to a methanol:ethanol 1.4:1 molar mixture. However, we believe that this does not affect the results, i.e., the observed trends are comparable, as the molecular simulations with a lower methanol ratio also showed the dominating behavior of methanol with its high concentration at the surface.

Again, we analyzed the density profiles at the different surfaces to provide molecular insight into the adsorption behavior. The density profiles of methanol at 100 °C at the nonpolar and polar surfaces in mixed systems is quite the same as the density profiles in the unmixed systems (see Figure S14). At 200 °C, there are some minor changes in the density profile of methanol molecules (see Figure S15a for the nonpolar surface and Figure S15b,c for the polar surfaces’ system). In this figure, the C1 density profile reveals a broad peak close to the nonpolar surface in comparison to the pure methanol system, where a sharp peak was found (Figure S11a). This suggests a stronger likelihood of the adsorption with the C1–O bond with a perpendicular/tilted configuration to the surface rather than in a parallel fashion (similar to the adsorption configuration shown in Figure S15d). Another difference that was observed is a shift between the second peaks of O and H(C) at the polar Zn surface, which occurred at the same distance from the surface in the methanol system. This suggests a change in the behavior of the second adsorbed layer by changing the adsorption concentration of methanol in the 1:1 methanol–ethanol mixture compared with the methanol system.

### Investigation of Other ZnO Precursors and Reaction Systems

According to the presented MD simulation results, the higher affinity of methanol to the polar ZnO surfaces (due to its higher polarity compared to ethanol) was confirmed, which clearly corroborates the experimentally observed retardation of the ZnO growth along the *c*-axis in the case of using pure methanol or even a methanol–ethanol solvent mixture. Thus, the higher affinity of methanol to the polar ZnO surfaces and its coordination to these surfaces inhibit the preferential growth of ZnO in the [001] direction, explaining the platelet-like ZnO structures obtained in methanol and the solvent mixture.

For a good correlation between the experimental and the simulation results, the experimental conditions were substantially simplified in order to exclude any factors that would not be represented in the simulations. Thus, the reaction system in the initial study was composed only of an anhydrous precursor and the alcohol solvent to be tested. At the same time, it has also to be taken into account that the counterion or organic moieties present in the precursor as well as side products of the synthesis could have an influence on the final morphology of the obtained product; this could not be included in the current simulation model, where only the affinity of the solvent to the different ZnO surfaces was investigated. As in the synthesis, it is impossible to use a ZnO precursor without a counterion or organic moiety, the precursor had to be carefully selected in order to minimize the influence of the counterion and achieve results that are experimentally representative of the insights gained from the MD simulations. ZnO precursors such as zinc acetylacetonate and zinc acetate result in the formation of ZnO without alkaline hydrolysis due to the presence of established Zn–O bonds within the precursor molecule. They have already been used in the literature for the synthesis of ZnO for multiple times.^4,9,49,50^

After performing the initial experiments using anhydrous Zn(acac)_2_ in a simple precursor-solvent system, further experiments were conducted using other precursors and reaction systems similar to those mostly found in the literature in order to correlate the ZnO growth observed in these systems with the model-supported findings in the simple alcohol systems. Table 2 shows an overview of literature results on the synthesis of ZnO in alcohols without the use of surfactants, revealing that mainly ZnO nanorods were obtained as products. It becomes clear from the table that the studies investigating methanol and ethanol as solvents mainly relied on an alkaline solvolysis where zinc acetate reacted with sodium hydroxide to form ZnO,^8,11,51^ except for Motelica et al.^7^ who used zinc acetate dihydrate as the precursor. No study previously investigated anhydrous Zn(acac)_2_ as a precursor in methanol and ethanol to compare the effect of these simple primary alcoholic solvents. To investigate the applicability of the insights gained above onto these systems, the precursors, Zn(acac)_2_ hydrate and zinc acetate as well as the alkaline hydrolysis with sodium hydroxide, are investigated.

**Table 2:** Overview of the Literature on the Synthesis of ZnO in Alcohols

precursor(s)	solvent	reaction conditions	product (primary particle morphology and aggregation behavior)	reference
Zn(OAc)_2_·2H_2_O + KOH	methanol	reflux for 24 h	ZnO nanorods; not aggregated	Pacholski et al.^52^
Zn(OAc)_2_·2H_2_O + NaOH	methanol, ethanol	150 °C for 24 h	ZnO nanorods of various aspect ratios	Cheng and Samulski^11^
Zn(OAc)_2_·2H_2_O	ethanol	100–200 °C for 24–48 h	ZnO nanoparticles (nearly spherical) and not aggregated	Du et al.^12^
Zn(OAc)_2_	1-butanol, 1-hexanol, 1-octanol, 1-decanol	250–300 °C for 2 h	ZnO nanorods of different aspect ratios; not aggregated	Ayudhya et al.;^6^ Tonto et al.^49^
Zn(acac)_2_	ethanol	120 °C for 20 h	ZnO nanorods aggregated forming a “radial morphology”	Xu et al.^10^
Zn(acac)_2_ hydrate	1-butanol, iso-butanol	reflux at 120–130 °C for 5 h	ZnO nanorods aggregated into bundles or complex “coral-like” structures	Ambrožiĉ et al.^38^
Zn(OAc)_2_·2H_2_O	methanol	60 °C for 3 h	spherical and elongaterd ZnO particles	Rajalakshmi et al.^13^
Zn(NO_3_)_2_·6H_2_O or ZnCl_2_ or Zn(OAc)_2_ + NaOH	methanol, ethanol	120 °C for 24 h	ZnO nanorods (smaller for methanol solvent and even spherical nanoparticles with ZnCl_2_ in methanol); no aggregate structures but some agglomeration	Rai et al.^51^
Zn(OAc)_2_·2H_2_O + KOH	ethanol	room temp. for 4 h	ZnO nanosheets aggregated in an “interwoven” structure	Khokhra et al.^42^
Zn(acac)_2_·H_2_O	ethanol, 1-propanol, 1-butanol, 1-pentanol, 1-octanol	170 °C for 4 h	ZnO nanorods except for 1-butanol where spherical primary particles are obtained; mainly spherical aggregates	Šarić et al.^9^
Zn(OAc)_2_·2H_2_O + NaOH	methanol, ethanol, butanol, hexanol, octanol, decanol	80 °C for 2 h, then room temp. for 24 h	spherical nanoparticles in methanol and decanol	Ramya et al.^8^
			mixed spherical nanoparticles and nanorods in ethanol and octanol	
			nanorods in butanol and hexanol ZnO nanoparticles and naorods are not aggregated.	
Zn(OAc)_2_·2H_2_O	methanol, ethanol, 1-propanol, 2-propanol, 1-butanol, 2-butanol, tert-butanol, 1-pentanol, 1-hexanol	60 °C for 24 h, then room temp. for 24 h	methanol: ZnO nanoparticles with “rounded shape” and low aggregation behavior	Motelica et al.^7^
			ethanol: ZnO nanoparticles forming “flower-like” aggregates	
			1-propanol, 1-butanol: “polyhedral” ZnO nanoparticles with some aggregation tendency	
			1-pentanol: ZnO nanoparticles of irregular shape without aggregation	
			1-hexanol: ZnO nanorods without aggregation	

### Synthesis from Zinc Acetylacetonate Hydrate and Zinc Acetate

In contrast to anhydrous Zn(acac)_2_, the hydrate form has been frequently used in the literature.^9,38^ In the case of the hydrate form, the water of crystallization will be released into the reaction system upon dissolution of the precursor, which thus could have an influence on the ZnO growth. Therefore, the difference between methanol and ethanol solvents for the ZnO formation from Zn(acac)_2_ hydrate was experimentally investigated.

In this case, also the XRD obtained for the different products synthesized at 100 and 200 °C showed the formation of ZnO with obvious differences in the peak intensity ratios (Figure S6a,b). The Scherrer equation was again applied to calculate the crystallite dimensions and corresponding aspect ratios to compare the particle morphology (Figure S6c,d). SEM images of the obtained products showed the presence of aggregates similar to the ones obtained from anhydrous Zn(acac)_2_; especially the ZnO product obtained in ethanol formed uniform spherical aggregates (Figure S7). Due to the strong aggregation behavior, mainly the results from XRD were used for evaluation of the primary particle shape. In general, it was found that slightly higher aspect ratios were obtained in comparison to anhydrous Zn(acac)_2_ (Figure 3) but also a similar trend was observed, where for ZnO synthesized at 200 °C in methanol aspect ratios near 1 were obtained, which is near to spherical. For ethanol, aspect ratios near 2 were obtained (nanorods), as shown in Figure S6d. The reason for the slightly higher aspect ratios obtained using Zn(acac)_2_ hydrate is most probably the water of crystallization being released into the reaction medium, leading to a more rapid growth of the ZnO nanocrystals, thus overshaping the role of the solvent. But still, it is clearly visible that methanol hinders the growth in the *c*-direction, resulting in nearly spherical nanoparticles in comparison to nanorods obtained using ethanol as a solvent. For the methanol–ethanol solvent mixture, the aspect ratios of the ZnO products obtained were also similar to using pure methanol as a solvent which again proves the dominating role of methanol on the ZnO growth.

**Figure 11:**
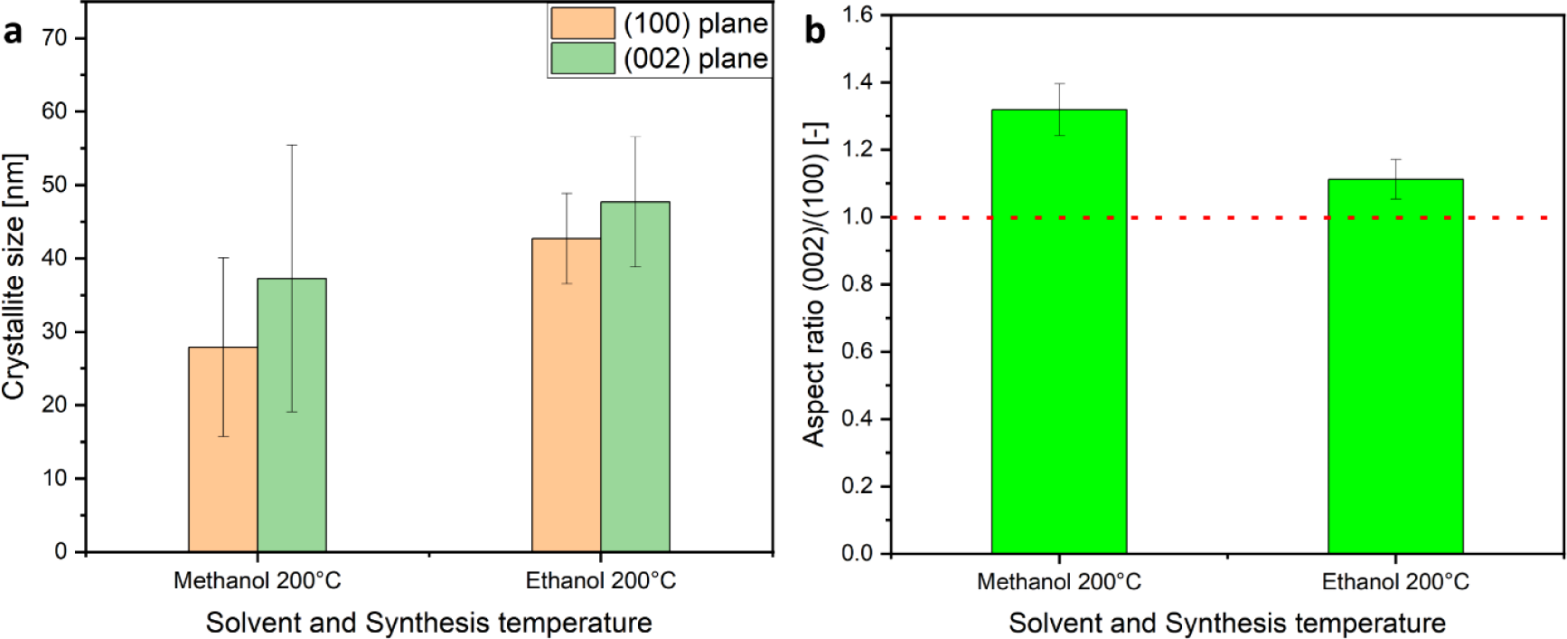
Bar chart showing (a) the crystallite size and (b) the corresponding aspect ratio calculated from the (100) and (002) XRD reflections for the products from ZnO syntheses at 200 °C using Zn(OAc)_2_ dihydrate as a precursor in methanol and ethanol.

In a further set of experiments, zinc acetate was selected as a precursor for ZnO synthesis. The reaction of the zinc acetate precursor with alcohols proceeds via an esterification reaction in the absence of water.^12,49^ Thus, using ethanol and methanol as solvents results in the formation of ethyl acetate and methyl acetate, respectively, as side products (see ^13^C NMR spectra in Figure S3c,d). Zinc acetate is present in an anhydrous form (anhydrous Zn(OAc)_2_) or as a dihydrate (Zn(OAc)_2_·2H_2_O). As in literature mainly the dihydrate was used,^7,12,13^ it was also tested here and shortly compared to the products obtained from the anhydrous form. The reaction was mainly conducted at 200 °C, as at 100 °C almost no product was formed. XRD patterns of the products (not shown) revealed the presence of wurtzite-phase ZnO as obtained from the other precursors. The crystallite size and the aspect ratio of the formed particles obtained via the Scherrer equation are shown in Figure 11. While in ethanol larger ZnO nanocrystals are formed than in methanol (Figure 11a) smaller aspect ratios (nearer to 1) were obtained in this case (Figure 11b). Similar observations were also made using anhydrous Zn(OAc)_2_ as the precursor (Figure S8).

**Figure 12:**
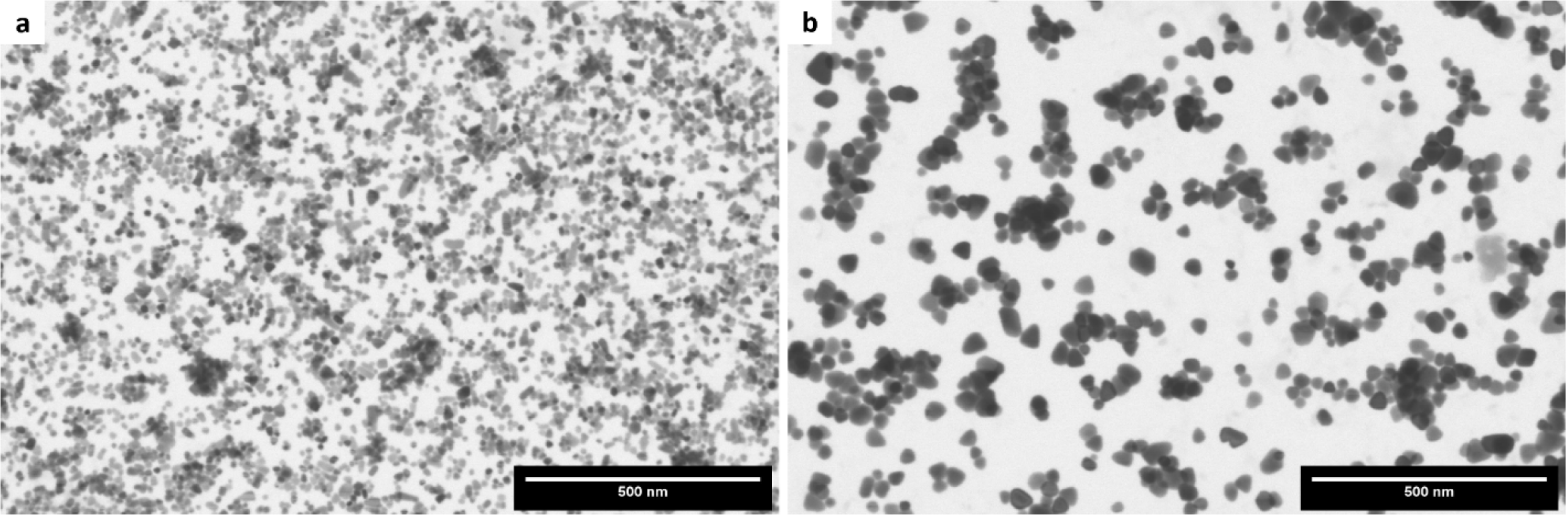
STEM images of the ZnO nanoparticles obtained from the precursor zinc acetate dihydrate in (a) methanol and (b) ethanol as solvents at 200 °C.

The difference in nanoparticle size could be due to the higher affinity or stronger binding of methanol to the different surfaces of ZnO as shown in the MD simulations (Figure 5) which results in slower growth kinetics in comparison to using ethanol as a solvent. But apparently, using zinc acetate as ZnO precursor resulted in ZnO nanoparticles with unaffected morphology regardless of the alcohol solvent used. This is also obvious in the STEM images taken for the particles obtained from the synthesis in methanol and ethanol (Figure 12) where only the different particle sizes along with a similar morphology is visible. Furthermore, in contrast to the zinc acetylacetonate precursors, nonaggregated particles were obtained in both solvents. Again, this shows the influence of the precursor type used and its organic moieties on the secondary structure of the final product. While the acetylacetonate precursor always resulted in aggregated products, the acetate precursor yielded nonaggregated particles. This can be also seen from the differences in the mass loss observed in the TGA for the products obtained from Zn(acac)_2_ and Zn(OAc)_2_ (Figure S5) where the greater mass loss observed for the product obtained from the Zn(acac)_2_ precursor points toward the presence of organics on the primary particles within the aggregates.

The difference in the growth behavior of the ZnO primary particles in comparison to the acetylacetonate products is also attributed to the organics in the precursor, with acetate as the counterion. As mentioned above, the reaction mechanism of ZnO formation is based on an esterification process when using alcohols as solvents, which also results in the in situ formation of water.^12^ Furthermore, acetate was found to be strongly binding to ZnO,^53^ which could strongly influence the ZnO formation as it will have the upper hand in determining the preferential growth directions of ZnO. This is also possibly the reason for the completely different aggregation behavior of the resultant ZnO nanocrystals, with the acetate on the particle surface resulting in particle stabilization and prevention of particle aggregation.

The role of the acetate species was further proven through a control experiment where anhydrous Zn(acac)_2_ was used as precursor while acetic acid was added to the reaction mixture to check whether the presence of acetate is responsible for the formation of nonaggregated particles. As expected, nonaggregated ZnO nanoparticles were formed (see Figure S9 as well as Figures S4 for IR spectra and S5 for TGA data of the product), with similar properties to those formed upon using zinc acetate as precursor. This significant role of acetate results in the decreased influence of the solvent (methanol or ethanol) in determining the resulting shape or aspect ratio of the resulting ZnO nanoparticles.

### Formation of ZnO in Alcohols Under Basic Conditions

**Figure 13:**
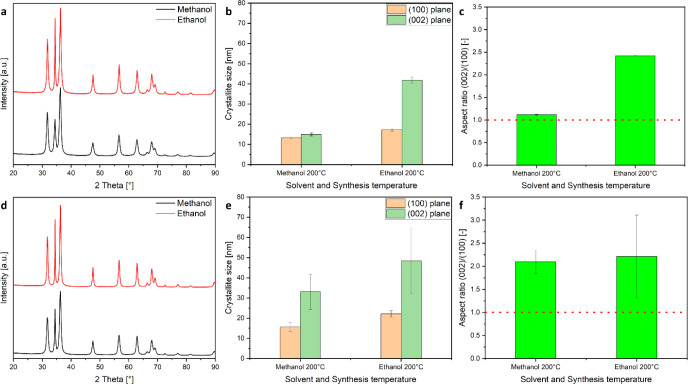
X-ray diffractograms of the ZnO products obtained from the synthesis using (a) Zn(acac)_2_ hydrate with NaOH and (d) Zn(OAc)_2_·2 H_2_O with NaOH as the precursor in pure methanol and pure ethanol at 200 °C. The corresponding bar charts show the crystallite size (b, e) and the aspect ratio (c, f) calculated using the Scherrer equation from the (100) and (002) reflections of the XRD measured for the products obtained from ZnO syntheses at 200 °C.

Another reaction for the formation of ZnO that is extensively used in literature is the synthesis under basic conditions.^8,11,42,51,52^ In this case, the presence of a base (usually NaOH or KOH) results in rapid decomposition of the ZnO precursor, whereby it is known that under basic conditions, the rate of the condensation step in the sol–gel process is increased, causing a rapid growth of ZnO nanocrystals. As a result, the influence of the solvent on the final particle morphology is reduced. Here, we investigated the reaction with NaOH in methanol or ethanol at 200 °C using the two precursors Zn(acac)_2_ hydrate or Zn(OAc)_2_·2H_2_O. The products obtained from both reactions in the different solvents were characterized via XRD, and the crystallite sizes as well as aspect ratios were calculated for comparison (Figure 13). In all cases, aspect ratios above 1 (nanorods) were obtained, which can be attributed to the more rapid growth kinetics caused by the base addition. But in case of using Zn(acac)_2_ hydrate in the alkaline solvolysis, a significant difference between the obtained aspect ratios for using methanol and ethanol as a solvent can be observed (Figure 13c). Much higher aspect ratios are achieved for the ZnO synthesized in ethanol in comparison with methanol, which can be attributed to the role of methanol hindering the growth in the [0001] or *c*-direction due to its higher affinity to the polar (0001) Zn surface, as outlined above. On the other hand, using Zn(OAc)_2_·2H_2_O as a precursor for this reaction suppressed the influence of the solvent on the aspect ratio due to the high affinity of acetate to the ZnO surface, with only a slight, nonsignificant difference remaining between the aspect ratios of the particles obtained from both solvents (Figure 13f). Hence, using a precursor that is devoid of counterions with high affinity to the ZnO and in the absence of any surfactants, the alcoholic solvent plays the dominant role in determining the structure and morphology of the resulting ZnO nanoparticles. In another work, Cheng and Samulski tuned the ZnO nanorod aspect ratio obtained from Zn(OAc)_2_·2H_2_O and NaOH by using methanol and ethanol as solvents, where the lower aspect ratios for the ZnO nanorods were found for the synthesis in methanol.^11^ The difference in our results can be attributed to the longer reaction times (24 h) and precursor concentrations (Zn(OAc)_2_·2H_2_O to NaOH ratio is 1:5) used but, on the other hand, correlates with the generally observed solvent effect in this work where methanol suppresses the *c*-directional ZnO growth.

## Conclusions

In this study, the morphological control of zinc oxide (ZnO) nanoparticles by a choice of simple primary alcoholic solvents was investigated. To this end, a simple model reaction system containing only the ZnO precursor and solvent was selected in order to avoid secondary influences as much as possible. The model reaction system involved the direct solvolysis of anhydrous zinc acetylacetonate in ethanol and methanol, where a remarkable dependence of morphology was unveiled; nanorod-shaped ZnO primary particles were formed in ethanol, while two-dimensional nanoplatelets were generated in methanol. Using a mixture of both solvents showed results similar to those using pure methanol as the solvent.

For getting insight into the solvent-dependent ZnO growth condition by molecular dynamics (MD) simulations, an interfacial force field was parametrized for describing the interaction of the ethanol|ZnO interface corresponding to the experimental conditions. The MD simulations revealed the preferential adsorption of methanol molecules onto the polar Zn-surface of ZnO in comparison to ethanol, which explains the “reversal” of the preferential growth direction of ZnO in the case of methanol. While in ethanol the *c*-direction is the preferential growth direction of ZnO, the growth along the *c*-axis is reduced in the case of using methanol as a solvent due to its preferential adsorption to the polar surfaces, resulting in a preferential growth in the *m*-direction. In the solvent mixture, a similar phenomenon is observed due to the higher affinity of the methanol molecules to the ZnO surfaces. The MD simulations, thus, offered a deeper understanding of the interactions at the atomic level that govern ZnO nanoparticle growth.

In order to evaluate the applicability of these findings to similar ZnO reaction systems often studied in the literature, the experimental study was extended to other precursors and reaction systems with addition of a base. These additional investigations allowed us to draw correlations and generalize the impact of solvents on ZnO nanoparticle morphology where the solvent influences—even if less pronounced—is still observed in these systems and a rational explanation for all resulting morphologies could be given.

In summary, this study highlights the crucial role of solvent selection in nanoparticle synthesis and design, focusing on the important aspect of morphology evolution in different reaction systems. This work, thus, contributes to a deeper understanding of the factors influencing the ZnO morphology, which is essential for the rational design and optimization of ZnO-based materials for various applications.

## Supporting Information Available

MD simulations: The optimized parameters interfacial methanol|ZnO and ethanol|ZnO force fields and comparison of calculated forces using the newly optimized ethanol|ZnO model with ab initio data, number of molecules in each simulated systems, number density profile of ethanol and methanol molecules at polar and nonpolar surfaces at 200 °C, and number density profile of methanol molecules in 1:1 methanol–ethanol mixture at 100 °C, experimental studies: ^13^C NMR spectra of the reaction mixtures, IR spectra of the ZnO products obtained from different precursors in ethanol, thermograms of the ZnO products obtained from different precursors in ethanol, XRD of the products obtained from the solvothermal synthesis using Zn(acac)_2_ hydrate as precursor, SEM images of the products obtained from the ZnO synthesis using Zn(acac)_2_ hydrate, and STEM image of the product obtained from anhydrous Zn(acac)_2_ with acetic acid (PDF)
