# Supplementary material for: Understanding the Role of Solvent on the Growth of Zinc Oxide: Insight from Experiment and Molecular Dynamics Simulations
Source: Langmuir. 2024 Sep 3;40(37):19343–56. doi: 10.1021/acs.langmuir.4c00921 (PMC11411714; doi:10.1021/acs.langmuir.4c00921)
Supplement: Supplementary file 1 — la4c00921_si_001.pdf [file la4c00921_si_001.pdf]

Supporting Information

Understanding the Role of Solvent on the Growth of Zinc Oxide: Insight from Experiment and Molecular Dynamics Simulations

Sherif Okeil[1,5], Sahar Rabet[2], Gerardo Valadez Huerta[3], Gabriele Raabe[2,], Georg Garnweitner[1,4,*]*

[1]Institute for Particle Technology, Technische Universität Braunschweig, Volkmaroder Str. 5, 38104 Braunschweig, Germany

[2] Institut für Thermodynamik, Technische Universität Braunschweig, Hans-Sommer-Str. 5, 38106 Braunschweig, Germany

[3] Center for Research Initiative for Supra-Materials, Shinshu University, 4-17-1 Wakasato, Nagano 380-8553, Japan

[4]Laboratory for Emerging Nanometrology, Technische Universität Braunschweig, Langer Kamp 6A, 38106 Braunschweig, Germany

[5]Pharmaceutical Analytical Chemistry Department, Faculty of Pharmacy, Ain Shams University, Abbassia, 11566 Cairo, Egypt

*g.garnweitner@tu-braunschweig.de, g.raabe@tu-braunschweig.de

Contents of Supporting Information

1. Parameters of the implemented Force Fields and calculated interfacial energies ……………………………………………………………………………… S-4

Table S1: Parameters of the methanol|ZnO interface Force Field based on the Ahlrichs-Penco-Scoles model.

Table S2: Parameters of the ethanol|ZnO interface Force Field based on the Ahlrichs-Penco-Scoles model.

Figure S1: Calculated interfacial forces fi for different ethanol configurations on the non-polar $(10\overline{1}0)$ surface.

Figure S2: Calculated interfacial energies for different ethanol configurations on the $(10\overline{1}0)$
Surface.

2. NMR Spectroscopy of the product suspensions after removal of solid products ……………………………………………………………………………... S-6

Figure S3: ^{13}C-NMR spectra of the reaction mixtures obtained after separating the formed ZnO through centrifugation from the reaction of (a) anhydrous $Zn(acac)_2$ with ethanol, (b) anhydrous $Zn(acac)_2$ with methanol, (c) $Zn(OAc)_2$ dihydrate with ethanol and (d) $Zn(OAc)_2$ dihydrate with methanol.

3. IR Spectroscopy of the ZnO particles ……………………………………………... S-7

Figure S4: IR spectra of the ZnO products obtained from different precursors in pure ethanol as solvent.

4. Thermogravimetric analysis of the ZnO products …………………………….. S-8

Figure S5: Thermograms of the ZnO products obtained from different precursors in pure ethanol as solvent.

5. XRD data and SEM images of ZnO nanoparticles from $Zn(acac)_2$ hydrate …. S-9

Figure S6: XRD of the products obtained from the solvothermal synthesis using $Zn(acac)_2$ hydrate as precursor in pure methanol, pure ethanol and a 1:1 (v/v) mixture of methanol and ethanol at (a) 100°C and (b) 200°C. Bar chart showing (c) the crystallite size and (d) the corresponding aspect ratio calculated using the Scherrer equation from the (100) and (002) reflexes of the XRD measured for the products obtained from ZnO syntheses at 100°C and 200°C using $Zn(acac)_2$ hydrate as precursor in the different solvents methanol, ethanol and the 1:1 methanol: ethanol solvent mixture.

Figure S7: SEM images of the products obtained from the ZnO synthesis using $Zn(acac)_2$ hydrate in (a) pure methanol, (b) a 1:1 v/v methanol-ethanol mixture and (c) pure ethanol at 200°C.

6. XRD data and SEM images of ZnO nanoparticles from anhydrous $Zn(OAc)_2$ S-11

Figure S8: Bar chart showing (a) the crystallite size and (b) the corresponding aspect ratio calculated using the Scherrer equation from the (100) and (002) reflections of the XRD measured for the products obtained from ZnO syntheses at 200°C using anhydrous $Zn(OAc)_2$ as a precursor in the different solvents methanol and ethanol.

Figure S9: STEM image of the product obtained from anhydrous $Zn(acac)_2$ with acetic acid in pure ethanol at 200°C.

7. Density profile of methanol on different ZnO surfaces ……………………….. S-12

Figure S10: Number density profiles of methanol molecules at (a) non-polar $(10\bar{1}0)$-ZnO and (b) polar (0001)-Zn and (c) the $(000\bar{1})$-O surfaces at 100℃.

Figure S11: Number density profiles of methanol molecules at (a) non-polar $(10\bar{1}0)$-ZnO and (b) polar (0001)-Zn and (c) the $(000\bar{1})$-O surfaces at 200℃.

8. Density profile of ethanol on different ZnO surfaces …………………………… S-15

Figure S12: Number density profiles of ethanol molecules at (a) the non-polar $(10\bar{1}0)$-ZnO, and (b) the polar (0001)-Zn and (c) the $(000\bar{1})$-O surfaces at 100℃.

Figure S13: Number density profiles of ethanol molecules at (a) non-polar $(10\bar{1}0)$-ZnO and (b) polar (0001)-Zn and (c) the $(000\bar{1})$-O surfaces at 200℃.

9. Density profile of methanol on different ZnO surfaces in 1:1 methanol-ethanol mixture ………………………………………………………………………………. S-18

Figure S14: Number density profiles of methanol molecules in 1:1 methanol-ethanol mixture at (a) non-polar $(10\bar{1}0)$-ZnO and (b) polar (0001)-Zn and (c) the $(000\bar{1})$-O surfaces at 100℃.

Figure S15: Number density profiles of methanol molecules in the 1:1 methanol-ethanol mixture at (a) the non-polar $(10\bar{1}0)$-ZnO and the polar (b) (0001)-Zn and (c) $(000\bar{1})$-O surfaces at 200℃.

1. Parameters of the implemented Force Fields and calculated interfacial energies

Table S1: Parameters of the methanol|ZnO interface Force Field based on the Ahlrichs-Penco-Scoles model

**APS-FF**	$\boldsymbol{A}$ in **eV**	$\boldsymbol{\rho}$ in Å	$\boldsymbol{rm}$ in Å	$\boldsymbol{C}$ in **eVÅ^6**	$\boldsymbol{D}$ in **eVÅ^8**	$\boldsymbol{E}$ **in eVÅ^{10}**
C-Zn	24226	0.2698	5.7078	1.88	0.2068	12.53
C- O_{ZnO}	291382	0.1026	1.5051	3.28	0.0	0.0
O-Zn	481	0.2115	2.0730	1.57	0.0	28.01
O-O_{ZnO}	2386	0.2896	3.5525	50.61	20.9641	0.31
HC-Zn	151934	0.1264	1.4493	3.90	0.9517	0.0
HC-O_{ZnO}	126236	0.1061	1.7543	6.74	3.1662	0.0
HO-Zn	206	0.3471	3.0155	33.27	0.0	607.72
HO-O_{ZnO}	735	0.2435	1.9242	15.09	1.0884	52.59

Table S2: Parameters of the ethanol|ZnO interface Force Field based on the Ahlrichs-Penco-Scoles model

**APS-FF**	$\boldsymbol{A}$ in **eV**	$\boldsymbol{\rho}$ in Å	$\boldsymbol{rm}$ in Å	$\boldsymbol{C}$ in **eVÅ^6**	$\boldsymbol{D}$ in **eVÅ^8**	$\boldsymbol{E}$ **in eVÅ^{10}**
C_1-Zn	21803	0.2742	4.5259	0.0	0.0	9077.82
C_1- O_{ZnO}	320520	0.0998	1.5055	3.27	0.0	0.0
C_2-Zn	23015	0.2718	4.9505	0.0	0.0	5299.26
C_2- O_{ZnO}	305951	0.1002	1.5048	3.27	0.0	0.0
O-Zn	433	0.2128	2.0761	1.61	0.0	24.48
O-O_{ZnO}	2147	0.2957	3.5862	44.14	145.09	150.42
HC_1-Zn	114225	0.1136	1.4466	3.18	0.0	12.17
HC_1-O_{ZnO}	119924	0.1259	1.7524	6.75	16264	7.58
HC_2-Zn	167127	0.1046	1.4506	4.2	0.0	0.0
HC_2-O_{ZnO}	138859	0.1230	1.7564	7.46	0.0	0.0
HO-Zn	226	0.3447	3.0298	33.71	0.0	729.76
HO-O_{ZnO}	730	0.2434	1.9299	15.13	0.1046	54.12

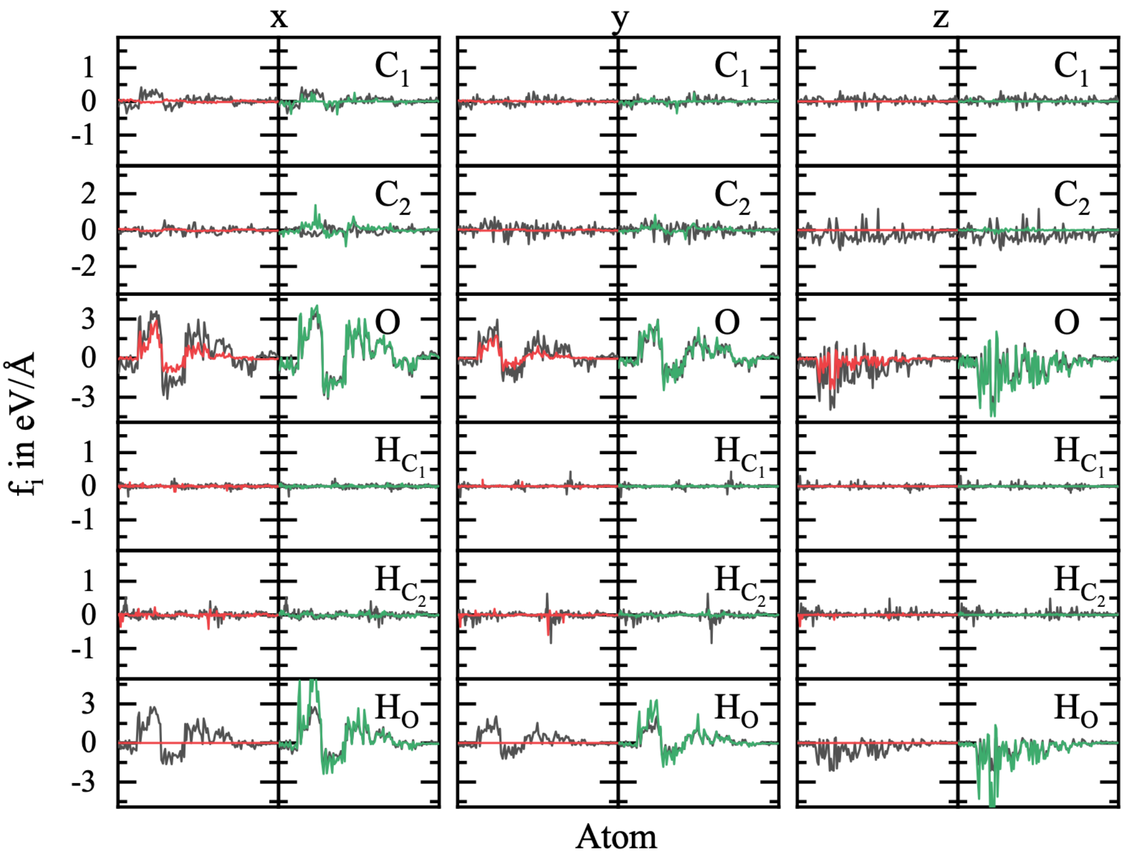

Figure S1: Calculated interfacial forces fi for different ethanol configurations on the non-polar $(10\bar{1}0)$ surface. Simulations results using the bulk force fields with standard combining rules (red line) and the ab initio based interface force field (green line) in comparison to ab initio data (black line).

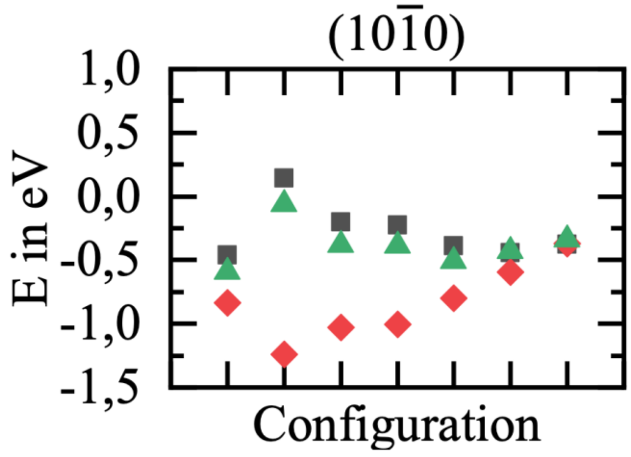

Figure S2: Calculated interfacial energies for different ethanol configurations on the $(10\bar{1}0)$ surface: simulations results using the bulk force fields with standard combining rules (red diamonds) and the ab initio based interface force field (green triangle) in comparison to ab initio data (black square).

Table S3: Number of molecules in each simulated system. For mixture systems the number is divided equally between two components, i.e., ethanol and methanol. * The number of molecules increased to 30% of original density ** The number of molecules increased to 10% of original density

Number of molecules in nonpolar slab		
	@ 100 °C	@ 200 °C
Methanol	1568*	1210*
Ethanol	842	654
1:1 molar Mixture	1090**	422**
Number of molecules in polar slab		
	@ 100 °C	@ 200 °C
Methanol	1183	914
Ethanol	826	641
1:1 molar Mixture	972	752

2. NMR Spectroscopy of the product suspensions after removal of solid products

^{13}C-NMR spectroscopy of the supernatant reaction mixture after removal of the ZnO nanoparticles through centrifugation was done to determine the side products formed during the reaction. For the measurement the reaction mixture was diluted in deuterated chloroform ($CDCl_3$) containing 0.03% TMS (Deutero GmbH) and transferred to an NMR tube (Wilmad 528-TR-7-V17M) to be measured on a Bruker Avance II 600 device at 151 MHz.

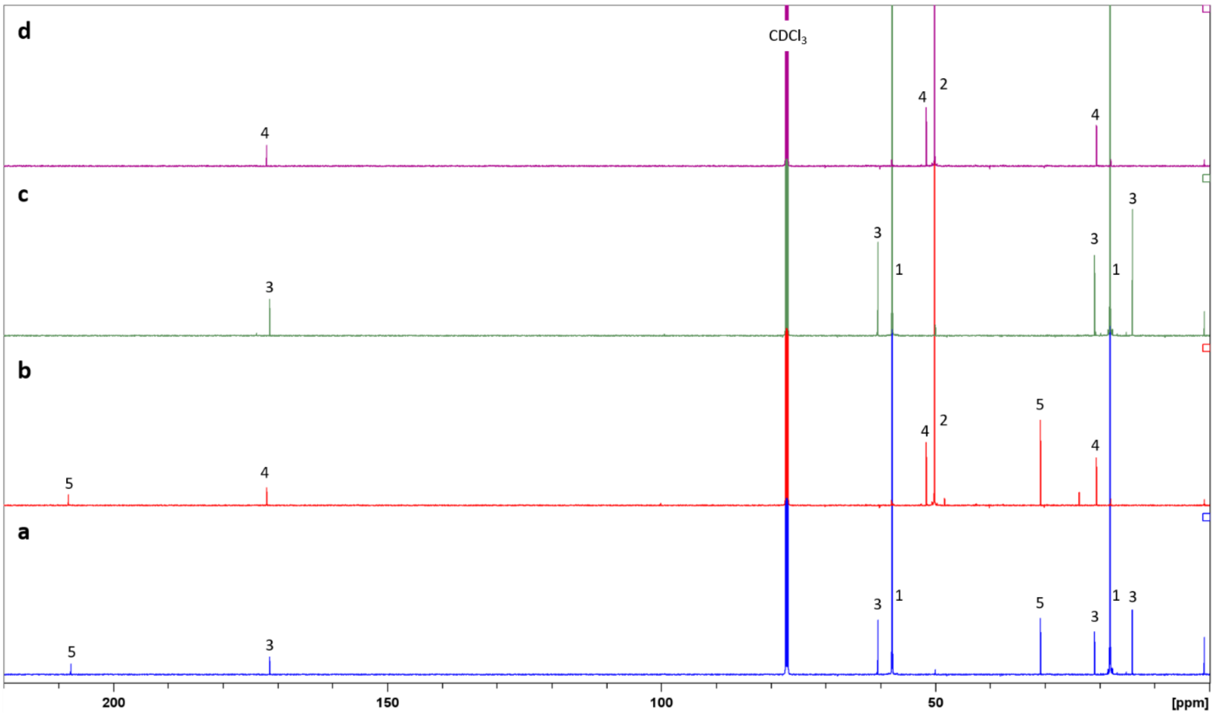

Figure S3: ^{13}C-NMR spectra of the reaction mixtures obtained after separating the formed ZnO through centrifugation from the reaction of (a) anhydrous $Zn(acac)_2$ with ethanol, (b) anhydrous

$Zn(acac)_2$ with methanol, (c) $Zn(OAc)_2$ dihydrate with ethanol and (d) $Zn(OAc)_2$ dihydrate with methanol. (1=ethanol, 2=methanol, 3=ethyl acetate, 4=methyl acetate and 5=acetone)

3. IR Spectroscopy of the ZnO particles

Fourier-transform infrared (FT-IR) measurements of the synthesized ZnO products were performed on a BRUKER Vertex V70 using an ATR crystal in the range of 4000 to 100 cm^{-1} with a resolution of 1 cm^{-1}.

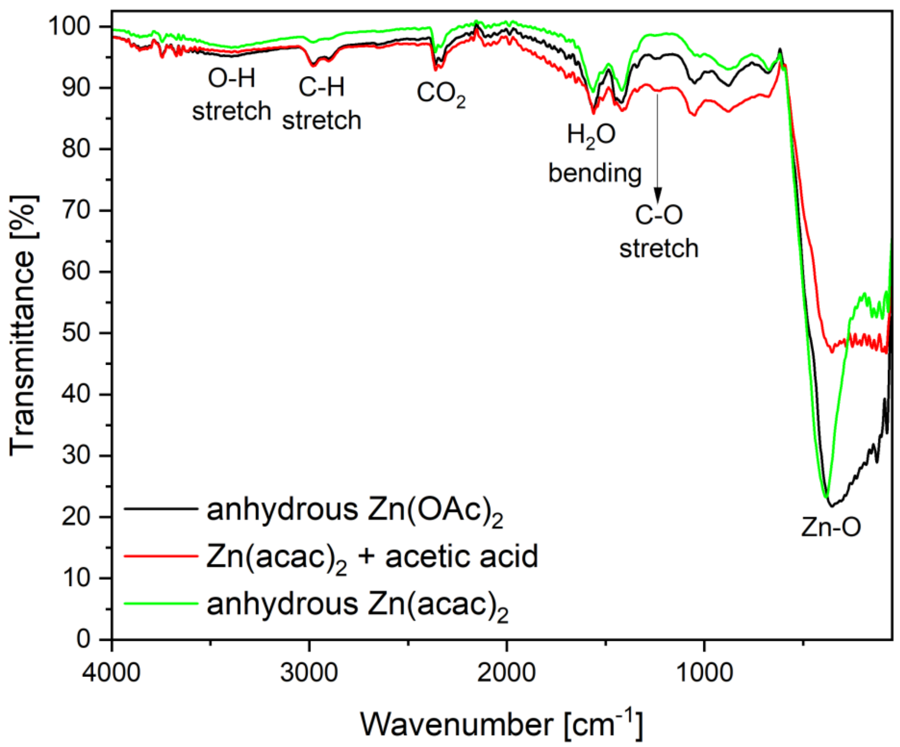

Figure S4: IR spectra of the ZnO products obtained from different precursors in pure ethanol as solvent.

4. Thermogravimetric analysis of the ZnO products

Thermogravimetric analysis (TGA) of the products was performed on a TGA/DSC 1 STARe system and a gas controller 4C200 STARe system from Mettler Toledo. For the TGA about 15 mg of sample was heated to 950°C under oxygen atmosphere with a heating rate of 10°C/min.

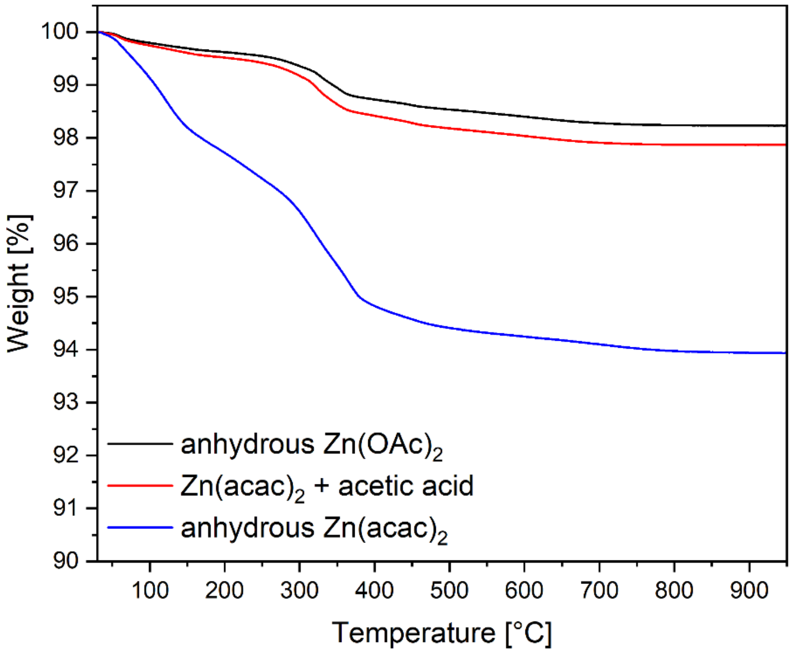

Figure S5: Thermograms of the ZnO products obtained from different precursors in pure ethanol as solvent.

5. XRD data and SEM images of ZnO nanoparticles from $Zn(acac)_2$ hydrate

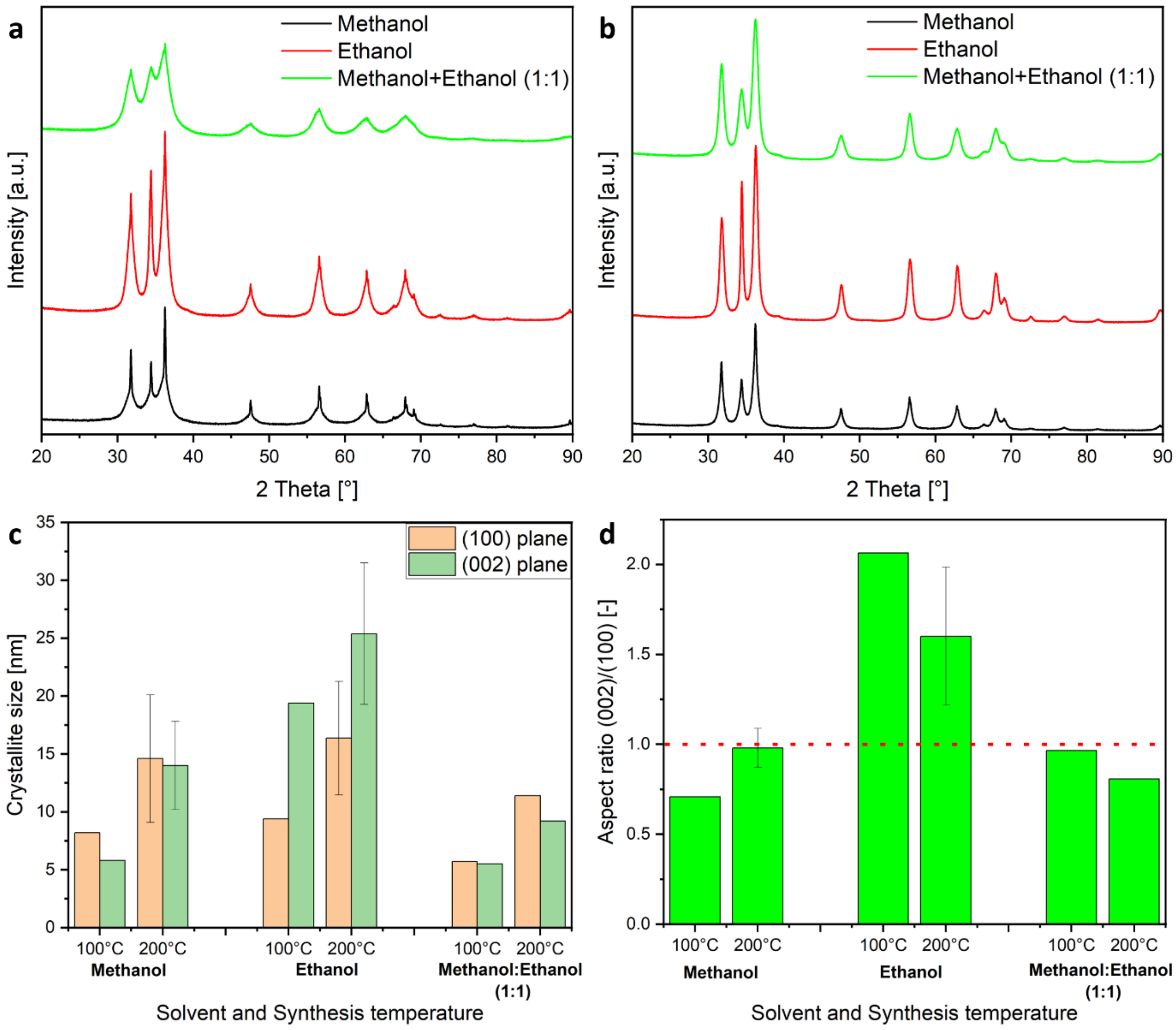

Figure S6: XRD of the products obtained from the solvothermal synthesis using $Zn(acac)_2$ hydrate as precursor in pure methanol, pure ethanol and a 1:1 (v/v) mixture of methanol and ethanol at (a) 100°C and (b) 200°C. Bar chart showing (c) the crystallite size and (d) the corresponding aspect ratio calculated using the Scherrer equation from the (100) and (002) reflexes of the XRD measured for the products obtained from ZnO syntheses at 100°C and 200°C using $Zn(acac)_2$ hydrate as precursor in the different solvents methanol, ethanol and the 1:1 methanol: ethanol solvent mixture.

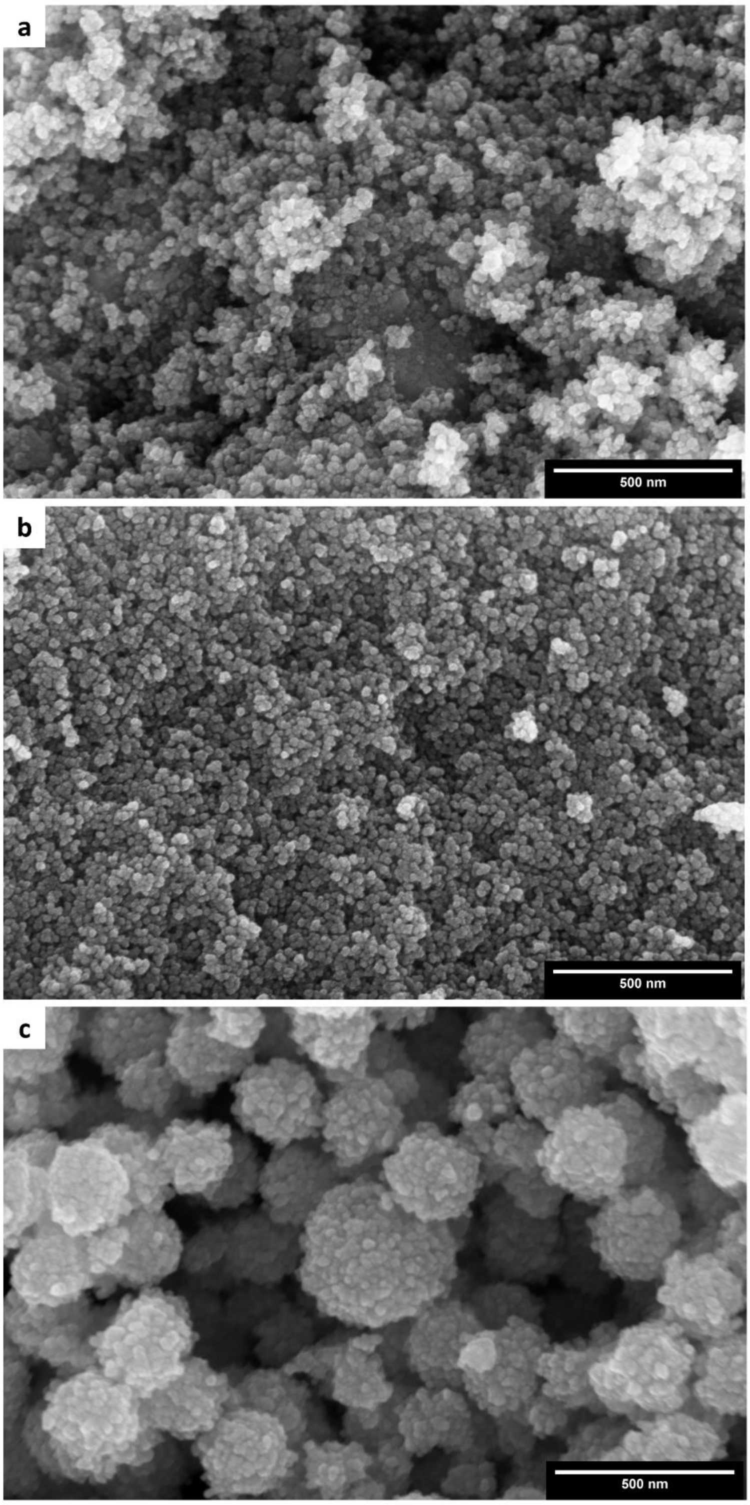

Figure S7: SEM images of the products obtained from the ZnO synthesis using $Zn(acac)_2$ hydrate in (a) pure methanol, (b) a 1:1 v/v methanol-ethanol mixture and (c) pure ethanol at 200°C.

6. XRD data and SEM images of ZnO nanoparticles from anhydrous $Zn(OAc)_2$

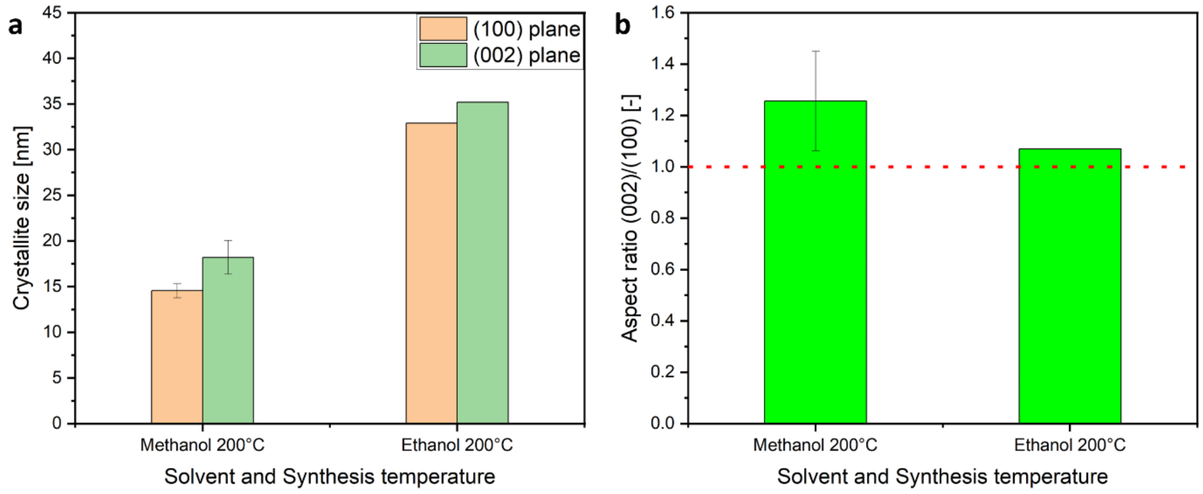

Figure S8: Bar chart showing (a) the crystallite size and (b) the corresponding aspect ratio calculated using the Scherrer equation from the (100) and (002) reflections of the XRD measured for the products obtained from ZnO syntheses at 200°C using anhydrous $Zn(OAc)_2$ as a precursor in the different solvents methanol and ethanol.

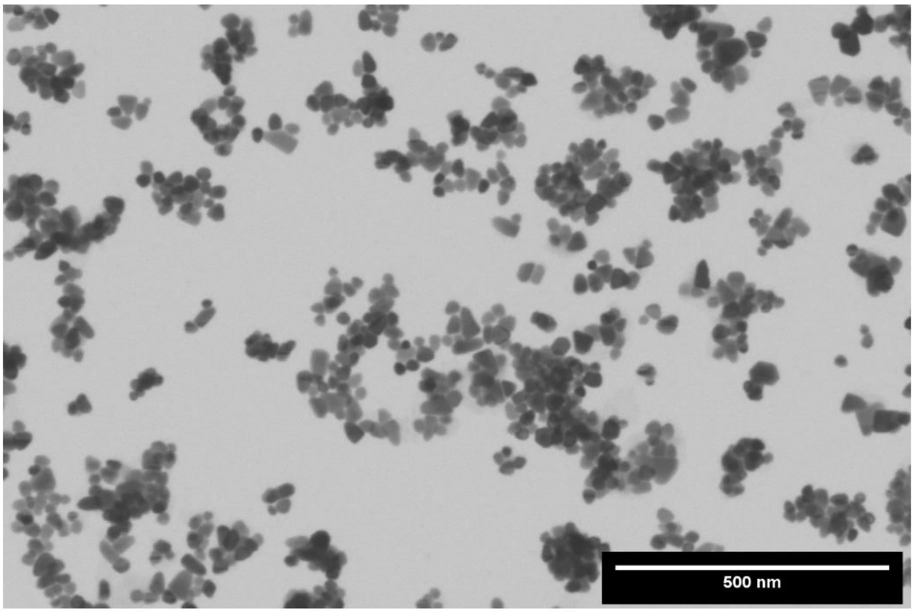

Figure S9: STEM image of the product obtained from anhydrous $Zn(acac)_2$ with acetic acid in pure ethanol at 200°C.

7. Density profile of methanol on different ZnO surfaces:

The number density profiles for methanol at the ZnO $(10\bar{1}0)$, (0001)-Zn, $(000\bar{1})$-O are shown in S10.

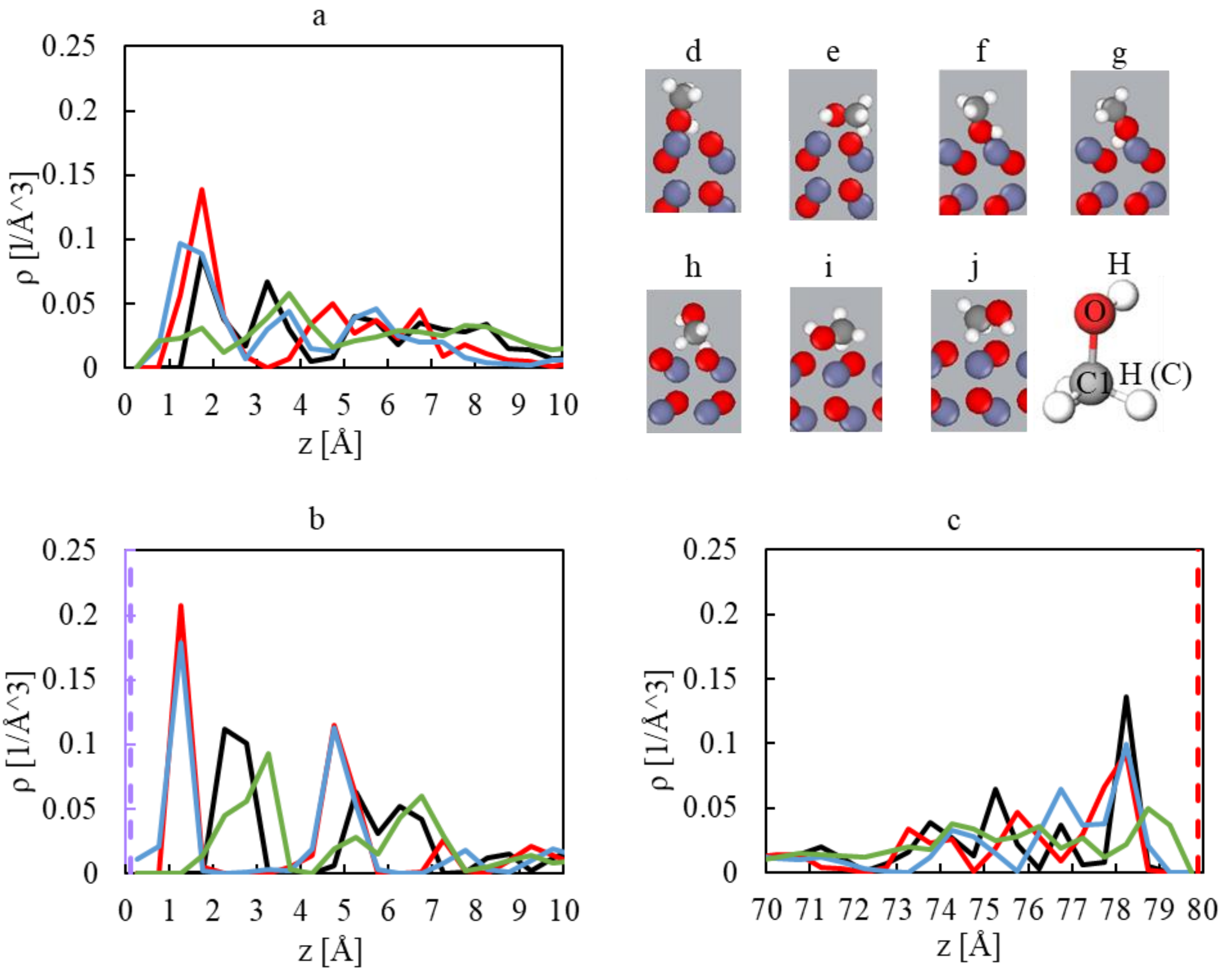

Figure S10: Number density profiles of methanol molecules at (a) non-polar $(10\bar{1}0)$-ZnO and (b) polar (0001)-Zn and (c) the $(000\bar{1})$-O surfaces at 100°C. In the polar systems, the left side is (0001)-Zn surface (purple dashed line) and right side is the $(000\bar{1})$-O surface (red dashed line). In each figure, the density of oxygen atom (O) is shown with red line, carbon (C1) with black line, hydrogen (H) in the hydroxyl group with blue and hydrogens (H(C)) in the methyl group with green line.

At non-polar surfaces (S10a), there is a peak of H atoms in the hydroxyl group at around 1 Å, followed by a higher peak of O, where the C1 density curve starts after the oxygen curve. These adsorption trends suggest that most of the molecules adsorbed with an orientation similar to the one shown in S10d, with the adsorption between the oppositely charged atoms Zn on the surface and O in methanol molecules, while the H atom in the hydroxyl group tends to form a hydrogen bond with the surface oxygen. This adsorption configuration was previously observed in the DFT studies to be the most stable[1,2]. As can be seen in this figure, a smaller peak of C1 at the same place as O is present. These two peaks, besides the small density of H(C) in methyl group at the close distance to the surface, suggest an adsorption configuration similar to S10e.

Although this configuration has a higher adsorption energy in the gas phase [2] than the most stable configuration, the interaction between the methanol molecules on the surface (as explained by Kiss et al.[1]) and the higher temperature in our study can influence the adsorption configurations. On the other hand, adsorption sites for the configuration in S10d may become occupied remaining only free sites for other adsorption configurations.

Figure S10b displays the density profile of methanol at (0001)-Zn polar surface. It shows two peaks of H and O that occur at the same distance from the surface, followed by a peak of C. This indicates a high inclination of the hydroxyl group to the surface as shown in S10f. There is a small density of H at the close distance to the surface (before the first peak) which can suggest a small number of molecules with the adsorption orientation similar to the S10g, in which the H in the hydroxyl group forms a hydrogen bond with the surface oxygen. In comparison to the non-polar surfaces, there is a clear trend of adsorption indicated by significantly higher H and O peaks. The oxygen peak approaches closer to the surface, which could be the result of strong adsorption between O and negatively charged Zn atoms at the surface. It can be seen in S10b that second peaks of H and O occur together at the same distance from the surface at about 5 Å. The strong orientation in the first layer and the interaction of O with the slightly positively charged methyl group between the first and second layers can cause this peak occurrence in the density profile curve and also in the concentration curve (Figure5b in the main document).

Figure S10c shows the density profile of methanol molecules at the polar $(000\bar{1})$-O surface. At this surface, the H(C) in the methyl group have a higher tendency towards the surface followed by a peak of carbon, which shows the higher tendency of methanol to adsorb to this surface with the methyl groups. We can assume the adsorption configuration to be similar to S10h or S10i or S10j. However, the H(C) peak is not significant and the peaks of C, O and H at the same distance from the surface indicated a higher probability of the adsorption configurations shown in 10i or S10j. The density profiles of the methanol molecules at the surfaces at 200°C possess a similar trend as at 100°C and are shown in Figure S11.

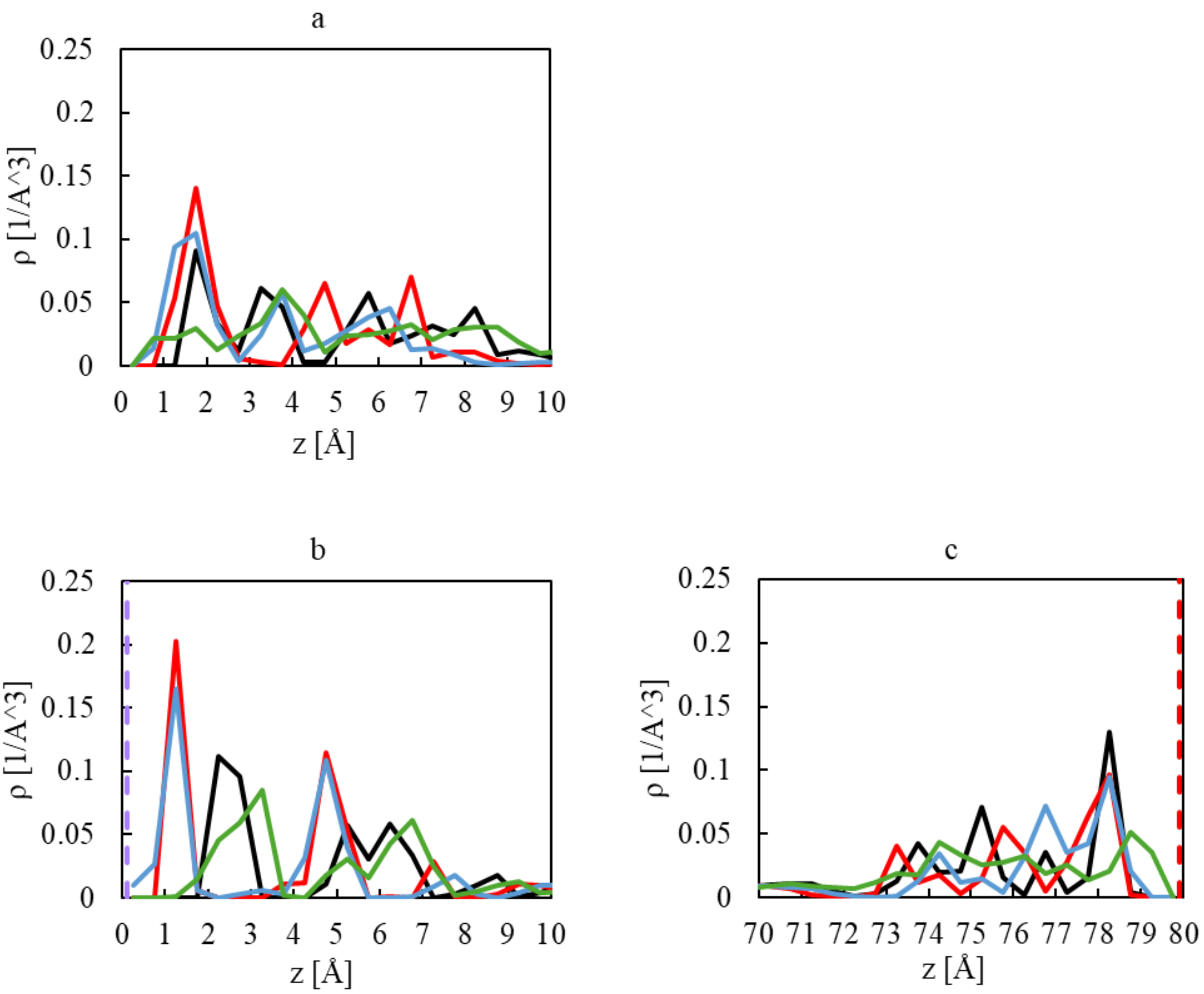

Figure S11: Number density profiles of methanol molecules at (a) non-polar $(10\bar{1}0)$-ZnO and (b) polar (0001)-Zn and (c) the $(000\bar{1})$-O surfaces at 200℃. In polar systems, the left side is (0001)-Zn surface (purple dashed line) and right side is the $(000\bar{1})$-O surface (red dashed line). In each figure, density of oxygen atom (O) shows with red line, carbon (C1) with black line, hydrogen (H) in hydroxyl group with blue and hydrogens (H(C)) in methyl group with green line.

8. Density profile of ethanol on different ZnO surfaces:

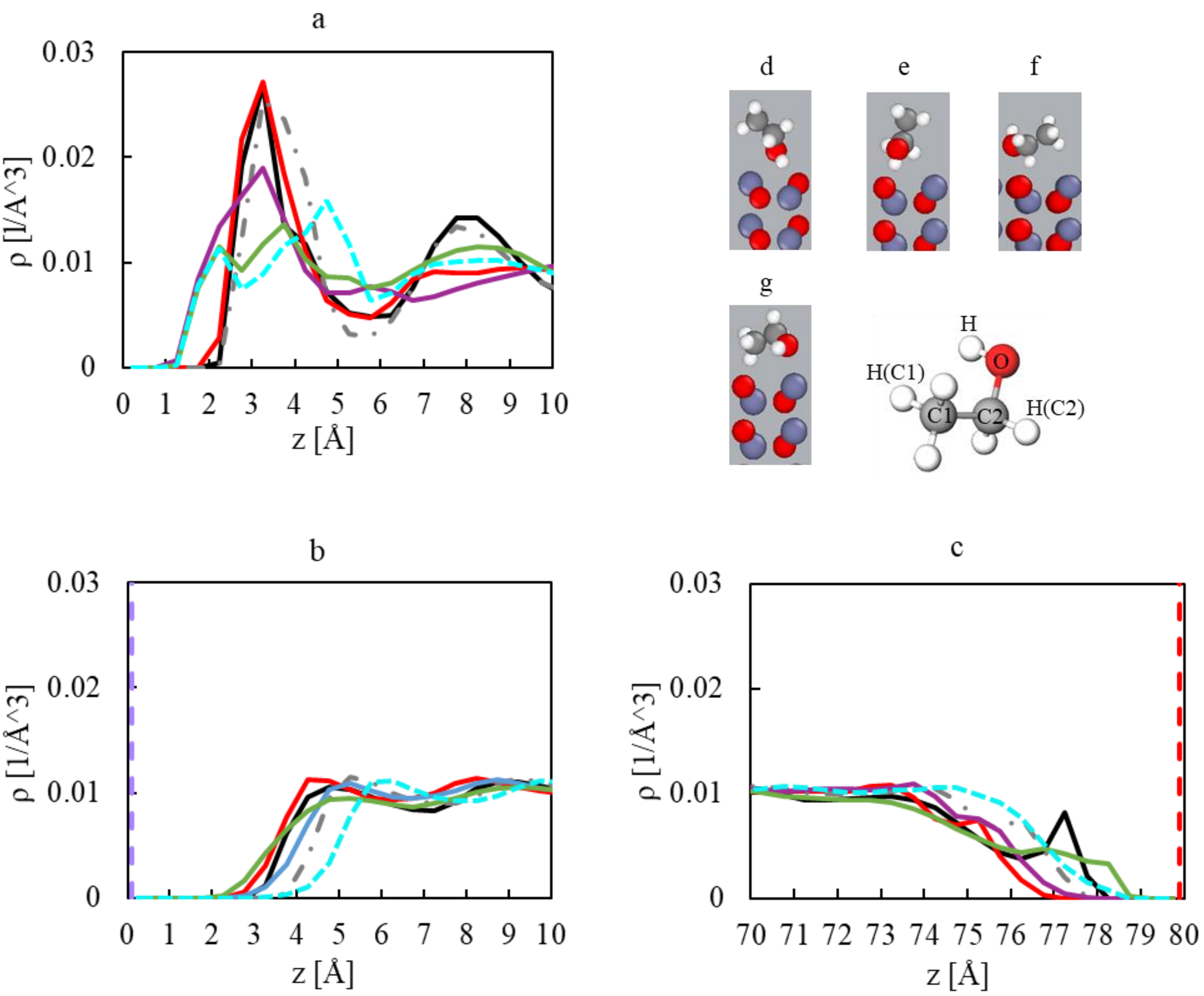

Figure S12: Number density profiles of ethanol molecules at (a) the non-polar $(10\bar{1}0)$-ZnO, and (b) the polar (0001)-Zn and (c) the $(000\bar{1})$-O surfaces at 100°C. In the systems with polar surfaces, the left side is the (0001)-Zn surface (purple dashed line) and the right side is the $(000\bar{1})$-O surface (red dashed line). In each figure, the density of oxygen atom (O) is shown with red line, carbon (C1) with black line, hydrogen (H) in the hydroxyl group with blue, hydrogens (H(C1)) in the methyl group with green line, the second carbon connected to the methyl group (C2) with dash-dotted grey line and hydrogen connected to this carbon (H(C2)) with dashed cyan line.

The number density profiles for ethanol at 100°C at the ZnO $(10\bar{1}0)$, ZnO (0001), ZnO $(000\bar{1})$ surfaces are depicted in Figure S12. As can be seen in Figure S12a, all hydrogen types (i.e. H, H(C1), H(C2)) are present in proximity to the non-polar surface. This behavior implies the presence of diverse adsorption configurations by approaching different hydrogens as shown in Figure S12d-g. However, the H atoms of the hydroxyl group show a higher density from 2Å up to a peak around 3Å. The higher density of hydrogen in hydroxyl group suggests a higher likelihood of adsorption with this functional group as illustrated in Figure S12d or Figure S12e. This adsorption orientation is almost similar to the most stable adsorption of ethanol determined

by DFT calculation.[3] However, as already mentioned the adsorption behavior is influenced by temperature, interaction between ethanol molecules and site competitiveness.

The adsorption trend of different atoms in the ethanol molecule at the (0001)-Zn surface is shown in Figure S12b. No substantial difference is observed in the adsorption densities of the different atoms. There is a low density of H(C1) and O available at a distance of about 3Å. However, it is not significant to suggest any specific adsorption configuration.

The density profile of ethanol molecules at the $(000\overline{1})$-O surface is depicted in Figure S12c, showing a minor peak of H (C1) present at about 2Å distance from the surface, followed by a peak for C1 at about 3Å. This suggests an adsorption trend with the methyl group oriented towards the surface. However, this peak is also not significant in comparison to other species. In general, the interaction of the ethanol molecule with the two polar surfaces is too weak to influence the orientation of the molecules on the surface, as shown in the concentration curve (Figure 8b). The density profiles of the ethanol molecules at the surfaces at 200°C have a similar trend as presented for 100°C and are shown in Figure S13.

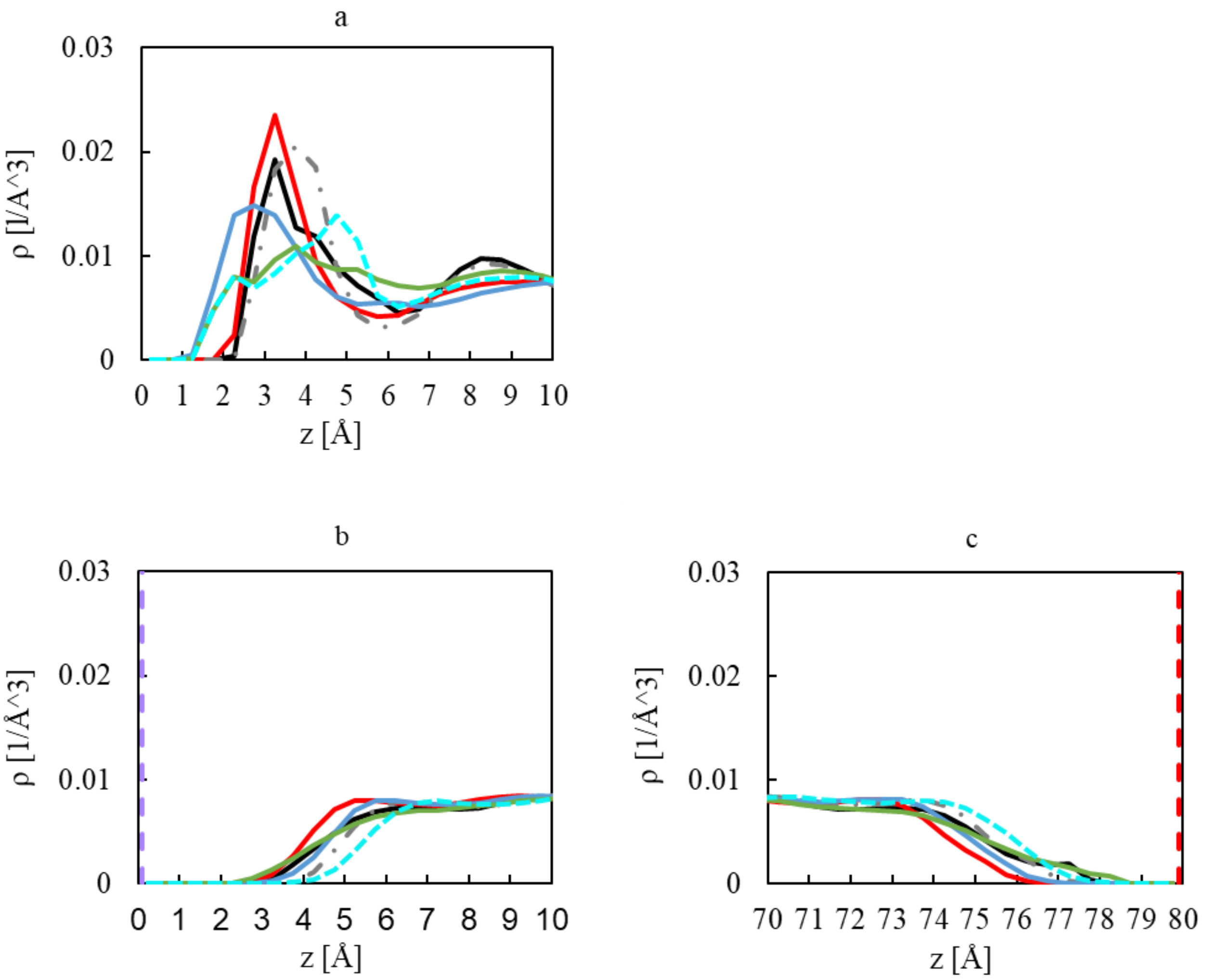

Figure S13: Number density profiles of ethanol molecules at (a) non-polar $(10\bar{1}0)$-ZnO and (b) polar (0001)-Zn and (c) the $(000\bar{1})$-O surfaces at 200°C. In polar systems, the left side is (0001)-Zn surface (purple dashed line) and right side is the $(000\bar{1})$-O surface (red dashed line). In each figure, density of oxygen atom (O) shows with red line, carbon (C1) with black line, hydrogen (H) in hydroxyl group with blue, hydrogens (H(C1)) in methyl group with green line, the second carbon connected to the methyl group (C2) with dash doted grey line and hydrogen connected to this carbon (H(C2)) with dashed cyan line.

9. Density profile of methanol on different ZnO surfaces in 1:1 methanol-ethanol mixture:

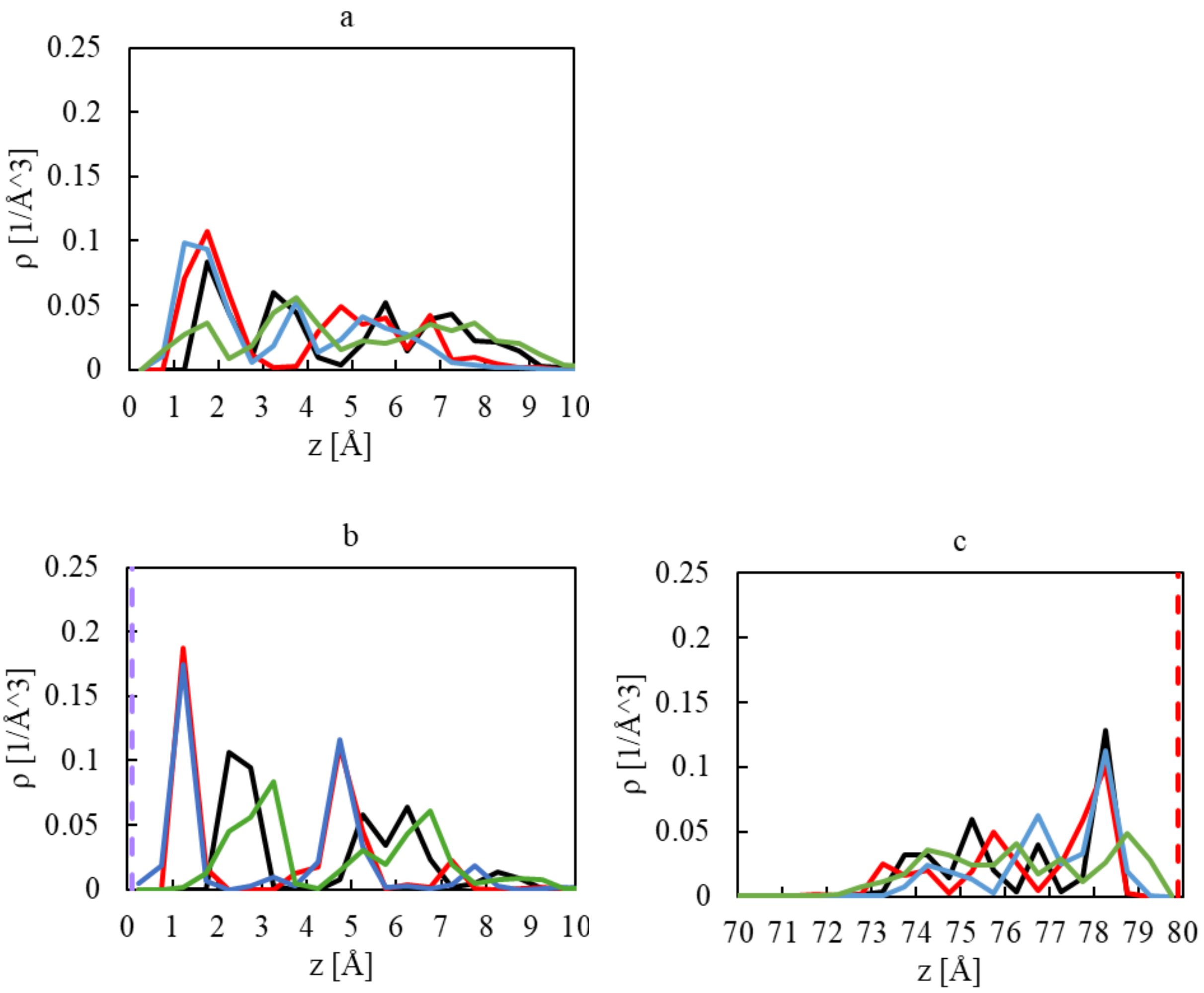

Figure S14: Number density profiles of methanol molecules in 1:1 methanol-ethanol mixture at (a) non-polar (10$\bar{1}$0)-ZnO and (b) polar (0001)-Zn and (c) the (000$\bar{1}$)-O surfaces at 100°C. In polar systems, the left side is (0001)-Zn surface (purple dashed line) and right side is the (000$\bar{1}$)-O surface (red dashed line). In each figure, density of oxygen atom (O) shows with red line, carbon (C1) with black line, hydrogen (H) in hydroxyl group with blue and hydrogens (H(C)) in methyl group with green line.

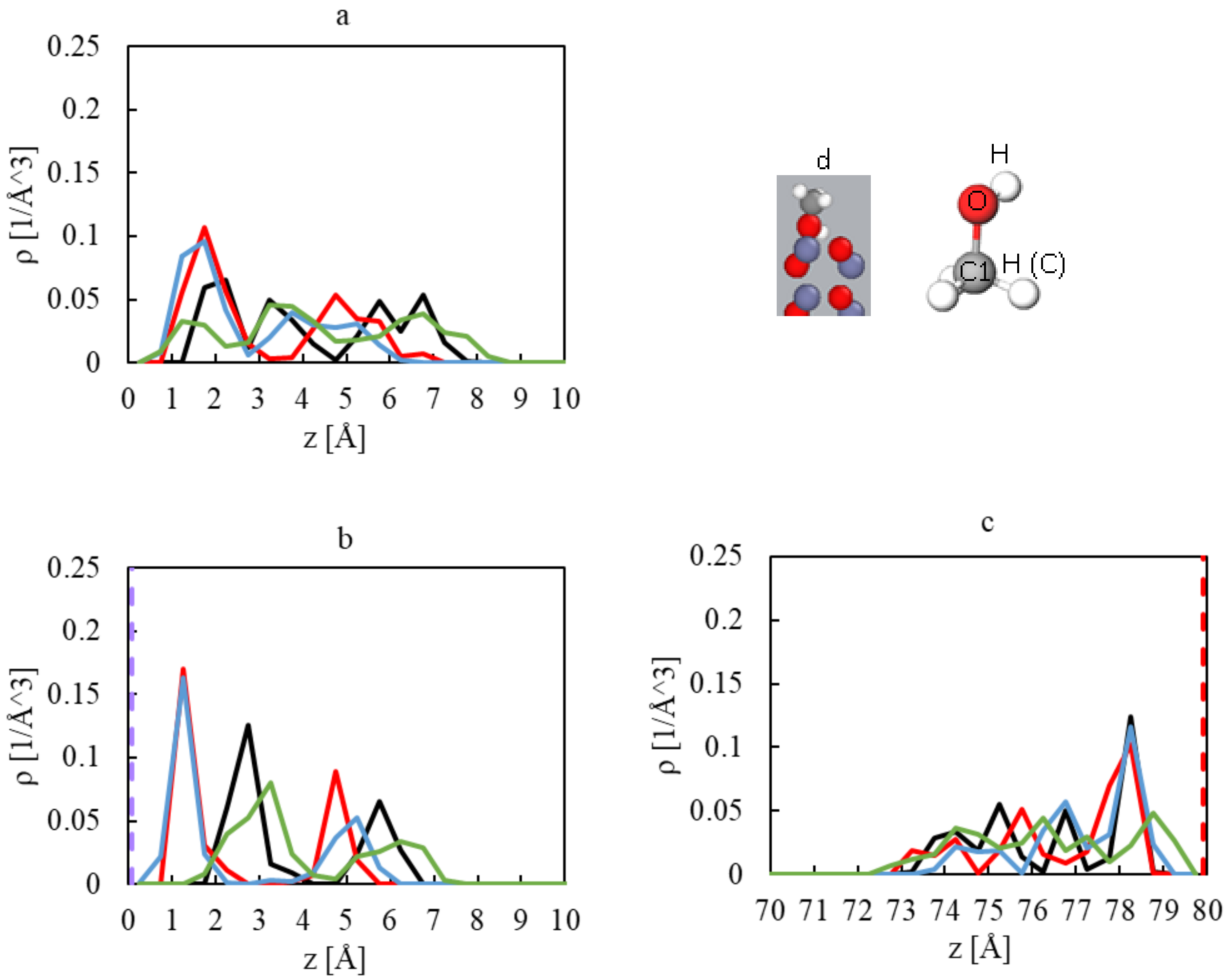

Figure S15: Number density profiles of methanol molecules in the 1:1 methanol-ethanol mixture at (a) the non-polar $(10\bar{1}0)$-ZnO and the polar (b) (0001)-Zn and (c) $(000\bar{1})$-O surfaces at 200°C. In the polar systems, the left side is (0001)-Zn surface (purple dashed line) and right side is the $(000\bar{1})$-O surface (red dashed line). In each figure, the density of oxygen atom (O) is shown with red line, carbon (C1) with black line, hydrogen (H) in hydroxyl group with blue and hydrogens (H(C)) in methyl group with green line.

References

(1) Kiss, J.; Langenberg, D.; Silber, D.; Traeger, F.; Jin, L.; Qiu, H.; Wang, Y.; Meyer, B.; Wöll, C. Combined Theoretical and Experimental Study on the Adsorption of Methanol on the ZnO(10$\bar{1}$0) Surface. *J. Phys. Chem. A* **2011**, *115* (25), 7180–7188. https://doi.org/10.1021/jp200146v.

(2) Vo, C. T.; Huynh, L. K.; Hung, J.-Y.; Jiang, J.-C. Methanol Adsorption and Decomposition on ZnO(101¯0) Surface: A Density Functional Theory Study. *Appl. Surf. Sci.* **2013**, *280*, 219–224. https://doi.org/10.1016/j.apsusc.2013.04.135.

(3) Yuan, Q.; Zhao, Y.-P.; Li, L.; Wang, T. Ab Initio Study of ZnO-Based Gas-Sensing Mechanisms: Surface Reconstruction and Charge Transfer. *J. Phys. Chem. C* **2009**, *113* (15), 6107–6113. https://doi.org/10.1021/jp810161j.
